# Supplementary material for: Shared genetic architectures of educational attainment in East Asian and European populations
Source: Nat Hum Behav. 2024 Jan 5;8(3):562–75. doi: 10.1038/s41562-023-01781-9 (PMC10963262; doi:10.1038/s41562-023-01781-9)
Supplement: Supplementary file 1 — Supplementary Note, Figs. 1–20, URLs and References. [file 41562_2023_1781_MOESM1_ESM.pdf]

nature human behaviour

Article https://doi.org/10.1038/s41562-023-01781-9

Shared genetic architectures of educational attainment in East Asian and European populations

In the format provided by the authors and unedited

Supplementary Information

Table of Contents

Supplementary Note **3**

1. Education system........ 3

1.1. Education system in Taiwan........ 3

1.2. Education system in Korea........ 4

2. Investigation of heterogeneity of genetic effects within EAS population........ 5

3. Polygenic prediction........ 6

3.1. Cohort description........ 6

3.2. Polygenic prediction under same sample size........ 8

Supplementary Figures **10**

Supplementary Figure 1. a. Quantile-Quantile (Q-Q) plot of GWAS for *EduYears* in TWB. b. Q-Q plot of GWAS for *EduYears* in KoGES. c. Q-Q plot of genome-wide meta-analysis for *EduYears* in East Asian. d. Q-Q plot of cross-population genome-wide meta-analysis for *EduYears* in East Asian and European.. 10

Supplementary Figure 2. a. Manhattan plot of GWAS for *EduYears* in TWB. b. Manhattan plot of GWAS for *EduYears* in KoGES. 11

Supplementary Figure 3. Manhattan plot of the heterogeneity for association of variants with *EduYears* between TWB and KoGES. 12

Supplementary Figure 4. The scatter plots of comparison for effect size of SNPs (BETA) between TWB and KoGES among SNPs with two-sided *P*-value $< 5 \times 10^{-8}$ in any one of the GWAS. a. Grouped by genome-wide significant level in TWB, KoGES, and genome-wide meta-analysis in East Asian........ 13

Supplementary Figure 5. Regional association plots 14

Supplementary Figure 6. The scatter plot of comparison for effect allele frequency between TWB and KoGES. 21

Supplementary Figure 7. a. Manhattan plot of GWAS for *EduYears* in the drinker group of KoGES. b. Manhattan plot of GWAS for *EduYears* in the non-drinker group of KoGES. 22

Supplementary Figure 8. SNP-based partitioned heritability in East Asian based on 97 functional genomic annotations. 23

Supplementary Figure 9. Tissue specific gene expression and chromatin enrichment in SNP-based heritability for *EduYears* in East Asian. 24

Supplementary Figure 10. Regional association plots for rs2881903 which was identified from EAS-specific cross-population meta-analysis using Multi-Ancestry Meta-Analysis (MAMA). 25

Supplementary Figure 11. Regional association plots for rs16930687 which was identified from EAS-specific cross-population meta-analysis using Multi-Ancestry Meta-Analysis (MAMA). 26
Supplementary Figure 12. Manhattan plot of Multi-Ancestry Meta-Analysis (MAMA) for *EduYears* in European. 27
Supplementary Figure 13. Cross- and within-population fine-mapping for *EduYears* in a genome-wide significant locus (rs10930013) in East Asian with *EduYears* GWAS in European by using SuSiEx. 28
Supplementary Figure 14. Cross- and within-population fine-mapping for *EduYears* in a genome-wide significant locus (rs2871133) in East Asian with *EduYears* GWAS in European by using SuSiEx.. 29
Supplementary Figure 15. Cross- and within-population fine-mapping for *EduYears* in a genome-wide significant locus (rs255347) in East Asian with *EduYears* GWAS in European by using SuSiEx. 30
Supplementary Figure 16. Cross- and within-population fine-mapping for *EduYears* in a genome-wide significant locus (rs7708343) in East Asian with *EduYears* GWAS in European by using SuSiEx. 31
Supplementary Figure 17. Cross- and within-population fine-mapping for *EduYears* in a genome-wide significant locus (rs16893804) in East Asian with *EduYears* GWAS in European by using SuSiEx. 32
Supplementary Figure 18. Cross- and within-population fine-mapping for *EduYears* in a genome-wide significant locus (rs11191157) in East Asian with *EduYears* GWAS in European by using SuSiEx. 33
Supplementary Figure 19. Scatter plot of the consistency between the GWAS summary statistics and the LD matrix from the reference panel 34
Supplementary Figure 20. Polygenic prediction of *EduYears* across discovery cohorts with consistent sample sizes in the EMCIT (*N* = 395; EAS), Korean-based cohort (N = 2,622; EAS), Chinese samples in the UKBB (*N* = 1,747; EAS) and the NIA-LOAD (*N* = 1,241; EUR) 41
URLs 42
Supplementary References 42

Supplementary Note

1. Education system

1.1. Education system in Taiwan

Education system in Taiwan includes six years of primary education, three years of junior high school, three years of senior secondary school, four years of higher education, one to four years for a master's degree, and two to seven years for a doctoral degree[1]. The academic year, which contains two semesters, will run from September 1st to August 31st of the following year. In the past, the Taiwan government announced a series of policies for the improvement in education level and education environment, such as compulsory education and equal education opportunity. 6-year compulsory education was announced in 1943, which forced children to be educated for six years of primary education when they aged six and above. In 1968, the Taiwan government extended 6-year compulsory education to 9-year compulsory education; for example, a person born on or after September 1955 had to go to elementary school for six years and junior high school for three years. In the current study, about one fifth of the TWB participants should follow the rule for 6-year compulsory education, and the remaining participants were regulated by law for 9-year compulsory education. A new policy for 12-year basic education[2], including 9-year compulsory education and 3-year popularization of high school education, was executed in 2014; however, this policy was not applied to participants in TWB.

1.2. Education system in Korea

In South Korea, the education system is based on a 6-3-3-4 system, consisting of six years of elementary school, three years of middle school, three years of high school, and four years of undergraduate university[3]. For degrees above this level, there are master's and doctoral degrees. Master's programs generally include two years and can be completed in as little as one year. A PhD, on the other hand, often takes three years or more, depending on the field and graduate school. All schools consist of two semesters, usually the first semester starts in March and the second semester begins at the end of August. The basic education system and master's and doctoral programs are structured in this way; however, compulsory education is designated under the law as a total of 9 years of education with 6 years of primary education and 3 years of secondary education. Compulsory education is 'education that systematically makes attendance at a certain school age compulsory' and has been widely implemented throughout the world in accordance with the idea of equal opportunity in education since the establishment of a modern nation[4]. In 1949, the 6-year compulsory elementary education law was regulated, but the policy was promoted in 1952 due to the Korean War. In 1984, a legal basis was established to expand compulsory education to middle school, and the policy was implemented from 1985[4]. In other words, the 6-year and 9-year compulsory education were executed in 1952 and 1985, respectively, and only 9-year education is compulsory in South Korea. In the current study, 64.6% of the KoGES participants were subject to 6-year compulsory education, and none of the participants had to follow 9-year compulsory education. This is because the year of birth of participants ranged from 1918 to 1973 in KoGES.

2. Investigation of heterogeneity of genetic effects within EAS population

To examine the factors contributing to the heterogeneity between TWB and KoGES near the *ALDH2* region on chromosome 12, we conducted a series of statistical analyses. Firstly, we calculated the Fst values between TWB and KoGES for the variants located in the *ALDH2* region, and we observed that the Fst values ranged from 4.12×10^{-8} to 0.045. When mapping the Fst values into percentiles across all loci that were included in EAS GWAS meta-analysis, 41 out of the 66 variants (62.1%) exhibited percentiles of 50% or higher. This finding suggests that the *ALDH2* region may possess divergent genetic structures, indicating potential genetic differences between TWB and KoGES populations. Next, we examined the PheWAS result in KoGES and estimated the global genetic correlation between alcohol drinking and *EduYears*, as described in the Methods section. To further estimate the local genetic correlation between alcohol drinking and *EduYears* in KoGES, we utilized the LAVA R package[5]. LAVA is a statistical framework capable of handling both continuous and binary phenotypes, enabling us to test local genetic correlation regardless of sample overlap. We estimated the sample overlap across traits using LDSC and incorporated these estimates as input for the LAVA analysis. To generate the locus definition file, which is a necessary input for the LAVA analysis, we utilized the partitioning algorithm provided by the LAVA framework. This algorithm allowed us to partition each chromosome into LD blocks. Specifically, we used the 1KG Project phase 3 EAS data and generated a total of 4,759 LD blocks. As a filtering step for the bivariate local genetic correlation analysis, we initially conducted univariate local genetic signal tests. Subsequently, we estimated bivariate local genetic correlation only in regions where a significant univariate genetic signal was observed, applying a significance threshold of P-value $< 0.05/4,759$. To account for multiple testing, we applied Bonferroni correction to determine the statistical significance of the estimated values of local genetic correlation.

3. Polygenic prediction

3.1. Cohort description

The Epidemiology of Mild Cognitive Impairment study in Taiwan (EMCIT)

The EMCIT is a prospective cohort study that investigated the epidemiology of mild cognitive impairment in community-dwelling older adults in Taiwan, which was established in 2017[6]. We obtained genome-wide genotype data from the TWB v2 custom array. For pre-imputation quality control (QC) and imputation, we used the same procedures as those used in our discovery samples.

The Korean-based cohort

The Korean-based cohort is a population-based cohort where data was collected between 2013 and 2019 and consists of 2,622 participants of Korean ancestry[7]. Participants were recruited from 20 referral hospitals in South Korea, including the Samsung Medical Center. The Illumina Asian Screening Array BeadChip (ASA Chip, Illumina, CA, USA) was used for genotyping. For the analysis, we restricted the variants that passed stringent QC criteria. We first filtered out variants based on the following criteria: (i) call rate < 98%, (ii) minor allele frequency (MAF) < 1%, and (iii) Hardy-Weinberg equilibrium (HWE) P-value < 10^{-6}. After variant-level QC, imputation was conducted using the Minimac4 software with Haplotype Reference Consortium (HRC-r1.1 2016) as the reference panel. Consequently, post-imputation QC involved filtering for variants with MAF < 1% and low imputation quality score (INFO) < 0.8. Based on genotype data, we excluded samples with (i) call rate < 95%, (ii) genetic and self-reported sex mismatch, (iii)

heterozygosity rates deviating by more than five standard deviations from the sample average, and (iv) identification as one of the related pairs with second-degree or closer relationships[7].

UK Biobank (UKBB)

The UKBB is a large prospective cohort with approximately 500,000 individuals across the United Kingdom. DNA samples were genotyped using the Affymetrix UK BiLEVE Axiom or Affymetrix UKB Axiom arrays (Santa Clara, CA, USA). We used imputed genome-wide genotype data provided by UKBB and filtered out the variants with the following criteria: (i) call rate < 95%, (ii) MAF < 0.5%, (iii) INFO < 0.4, and (iv) HWE P-value < 10^{-6}. To classify Chinese ancestry among these individuals, we applied the method of ancestry grouping described by Privé *et al.*[8], which used internal PC scores and self-reported ethnic background. We obtained data provided by Privé *et al.*[8], which defined centres of self-reported ethnic background (Data-Field 21000) using the UKBB data. Then, the ancestral group was inferred through the process of matching all individuals to one of these centres.

The National Institute on Aging Genetics Initiative for Late-Onset Alzheimer's Disease (NIA-LOAD)

NIA-LOAD[9,10] is a cohort of 1,877 individuals from families with late-onset Alzheimer's disease, as well as unrelated and non-demented individuals. For genotyping, the Illumina Infinium II assay protocol with hybridisation to Illumina Human 610Quadv1_B BeadChips was used. Before imputation, we performed QC at both the variant and sample levels. For the variant-level QC, we applied the following criteria: (i) call rate > 98%, (ii) MAF > 1%, and (iii) HWE P-value < 10^{-6}. Subsequently, we removed samples exhibiting inconsistencies between genetic and

self-reported sex, as well as samples with heterozygosity rates deviating from the sample average by more than five standard deviations. For the imputation, we used the HRC-r1.1 2016 as the reference panel, and then filtered out variants with MAF < 0.5% and INFO < 0.8.

3.2. Polygenic prediction under same sample size

We conducted additional association and PGS analyses by sub-sampling data from the EAS and EUR (UKBB) cohorts for *EduYears* in four settings: (1) EUR (n = 176,400), (2) EUR (n = 352,800), (3) cross-ancestry GWAS (n = 176,400; EAS [n = 88,200] + EUR [n = 88,200]), and (4) cross-ancestry GWAS (n = 352,800; EAS [n = 176,400] + EUR [n = 176,400]). The sub-cohorts for the EAS population were generated from TWB, while sub-cohorts for the EUR population were extracted from the UKBB. As described in the Methods section, we constructed PGSs for *EduYears* using PRS-CS[11] and PRS-CSx[12], and their ability was evaluated in four independent testing cohorts, including the EMCIT, a Korean-based cohort, the Chinese samples in UKBB, and the NIA-LOAD. When the sample size was set to 176,400, the cross-population PGS for *EduYears* explained up to 1.8% of the phenotypic variance in *EduYears* in the EAS cohorts, whereas the EUR PGS explained up to 1.0% of the phenotypic variance. Notably, when the sample size was adjusted to 357,800, the cross-population PGS for *EduYears* exhibited a significantly enhanced predictive performance compared to the EUR PGS. The EUR PGS explained only up to 1.4% of the phenotypic variance in *EduYears* in the EAS cohorts, while the cross-population PGS accounted for up to 4.0% of the phenotypic variance. However, across all settings, the EUR PGS showed better performance in the NIA-LOAD, which is an ancestry-matched target cohort of EUR ancestry. In conclusion, our PGS analyses under the same sample size demonstrated that the cross-population PGS consistently explained a greater proportion of the phenotypic variance in *EduYears* compared to the EUR PGS in the EAS target cohorts.

Additionally, the performance improvement was evident with larger sample size. These results highlight the importance of not only increasing sample sizes but also including population diversity to enhance the predictive performance of PGSs.

Supplementary Figures

Supplementary Figure 1. a. Quantile-Quantile (Q-Q) plot of GWAS for *EduYears* in TWB. b. Q-Q plot of GWAS for *EduYears* in KoGES. c. Q-Q plot of genome-wide meta-analysis for *EduYears* in East Asian. d. Q-Q plot of cross-population genome-wide meta-analysis for *EduYears* in East Asian and European. The *x* axis represents the expected value of $-\log_{10}(P\text{-value})$ for association of variants with *EduYears*, and the *y* axis represents the observed value of $-\log_{10}(P\text{-value})$ for association of variants with *EduYears*. All *P*-values were calculated by a two-sided test. The red line marks the diagonal line.

a

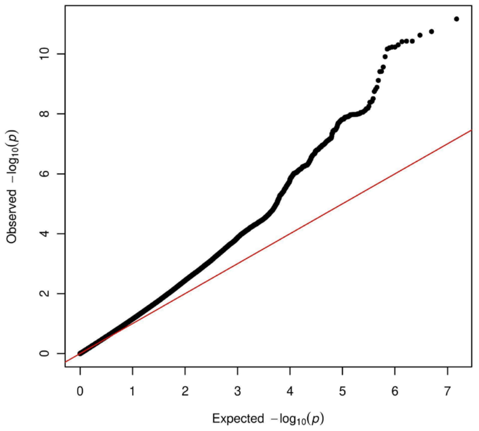

b

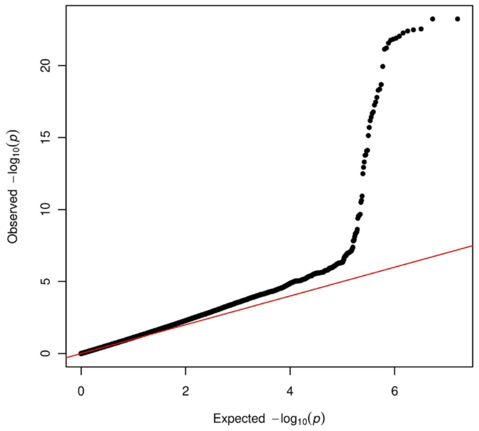

c

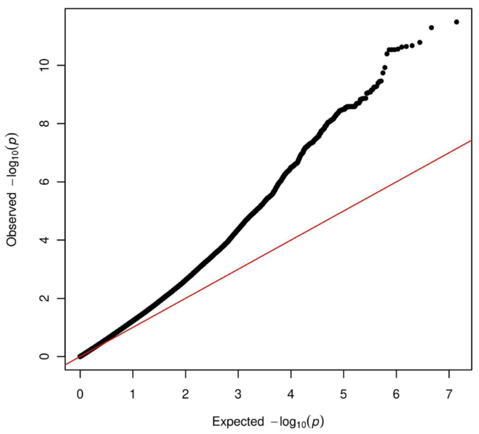

d

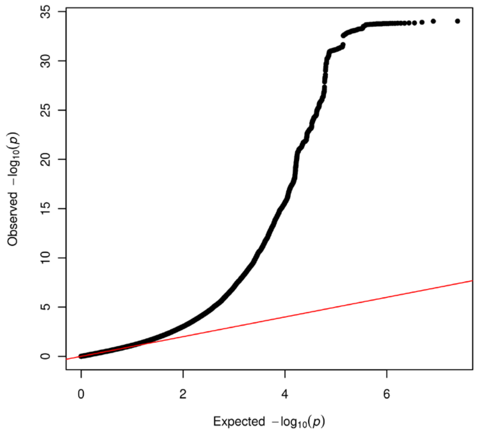

Supplementary Figure 2. a. Manhattan plot of GWAS for *EduYears* in TWB. b. Manhattan plot of GWAS for *EduYears* in KoGES. The x axis represents chromosomal position, and the y axis represents the $-\log_{10}$(P-value) for association of variants with *EduYears*. Reported P-values are two-sided and not corrected for multiple testing. Independent SNPs are highlighted in green, and novel SNPs are highlighted as a red diamond. The horizontal pink line marks the threshold for genome-wide significance (P-value = 5×10^{-8}), and the horizontal blue line marks the threshold for suggestive genome-wide significance (P-value = 1×10^{-5}).

a

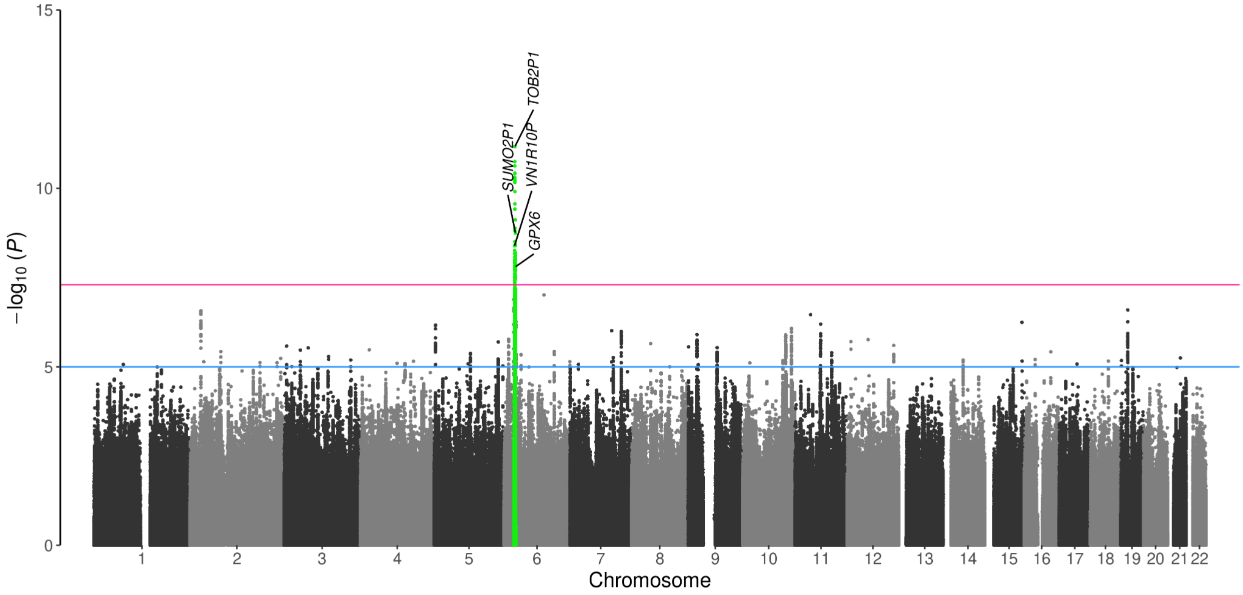

b

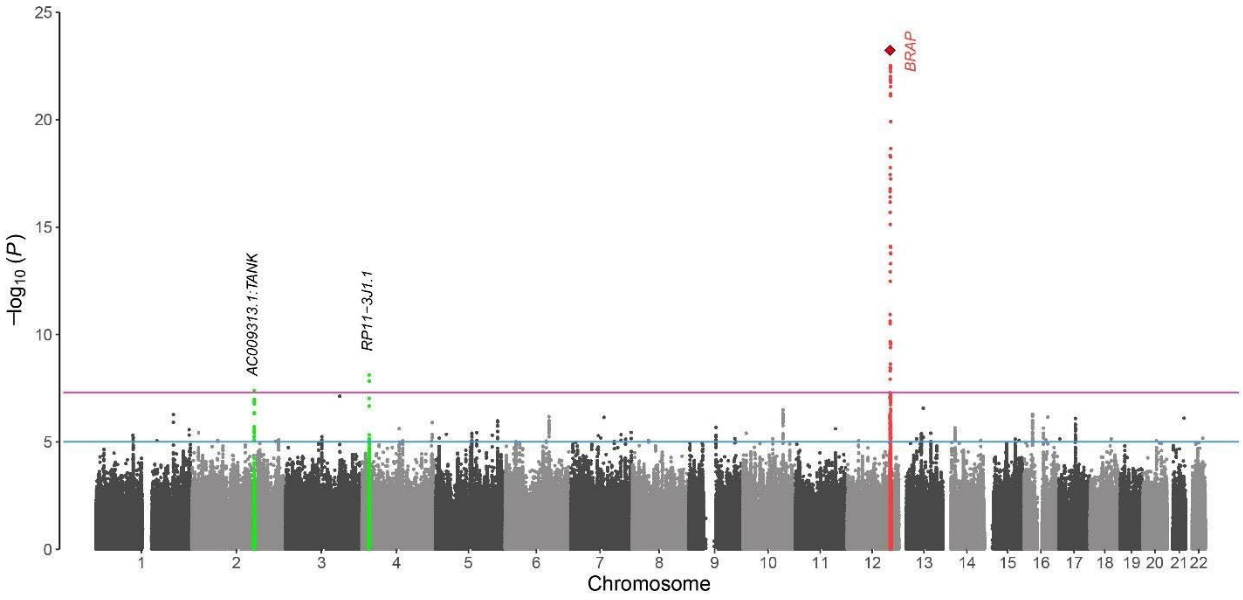

Supplementary Figure 3. Manhattan plot of the heterogeneity for association of variants with *EduYears* between TWB and KoGES. The *x* axis represents chromosomal position, and the *y* axis represents the $-\log_{10}$(P-value) for heterogeneity of association of variants with *EduYears* between TWB and KoGES. All P-values are two-sided. The horizontal red line marks the threshold for genome-wide significance (P-value = 5×10^{-8}), and the horizontal blue line marks the threshold for suggestive genome-wide significance (P-value = 1×10^{-5}).

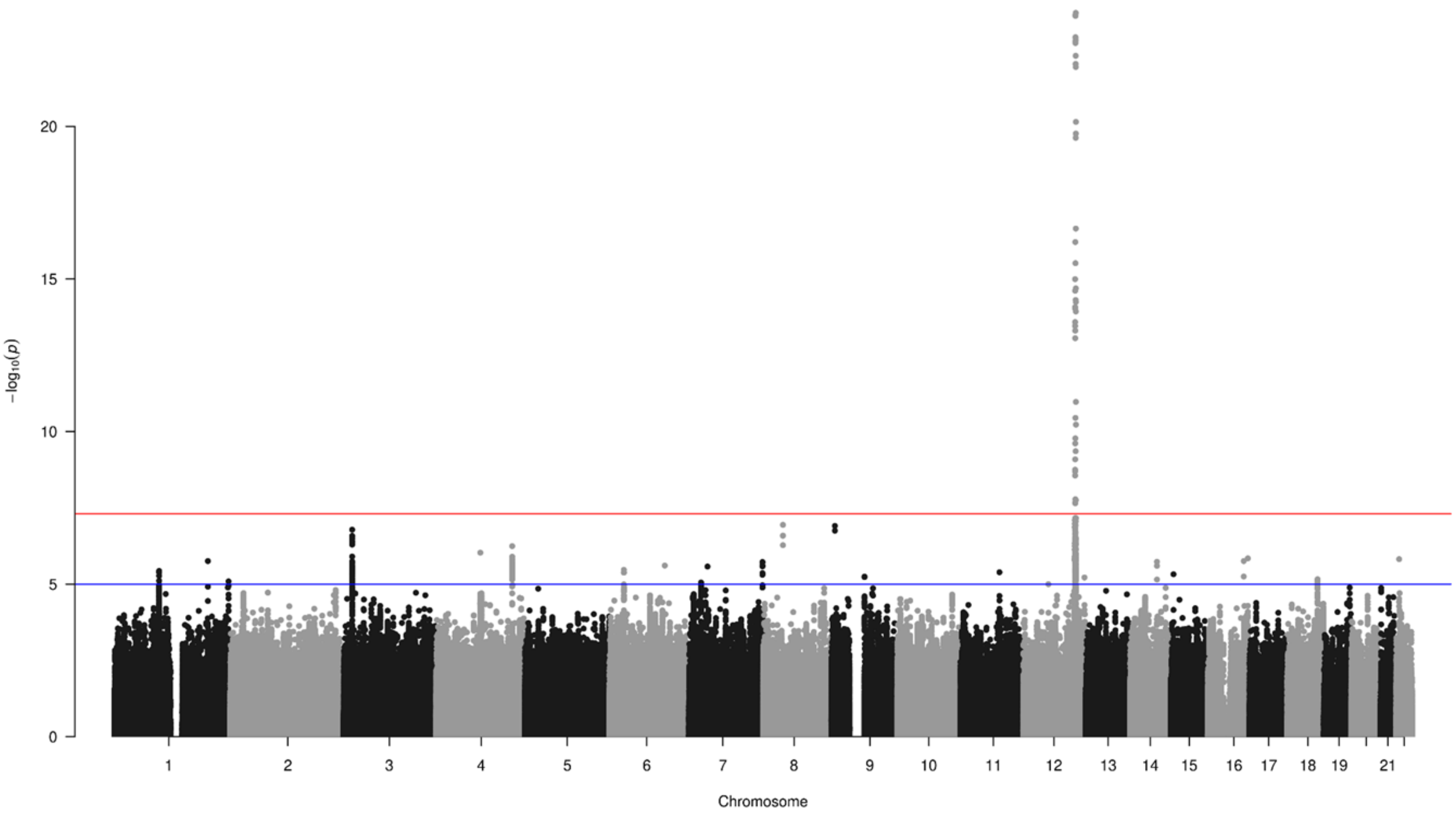

Supplementary Figure 4. The scatter plots of comparison for effect size of SNPs (BETA) between TWB and KoGES among SNPs with two-sided *P*-value < 5×10^{-8} in any one of the GWAS. a. Grouped by genome-wide significant level in TWB, KoGES, and genome-wide meta-analysis in East Asian. Green, blue, and red points are SNPs that passed genome-wide significance in only one GWAS. Yellow and pink points are SNPs that passed genome-wide significance in one of TWB and KoGES as well as in genome-wide meta-analysis in East Asian. **b. Grouped by chromosome 12 and others.** SNPs on chromosome 12 are highlighted in red. For both panel a and b, the *x* axis represents effect size of SNPs in TWB, and the *y* axis represents effect size of SNPs in KoGES. Each point represents the effect size of an identified SNP that passed genome-wide significant *P*-value threshold of a two-sided test in any one of the GWAS in TWB, KoGES, and genome-wide meta-analysis in East Asian. Points lie on the diagonal line indicating the effect size of SNPs are identical between TWB and KoGES. Points lie on the first and third quadrants which indicate that the direction of effect size is consistent between TWB and KoGES; in contrast, points lie on the second and fourth quadrants which indicate that the direction of effect size in TWB is opposite to the one in KoGES.

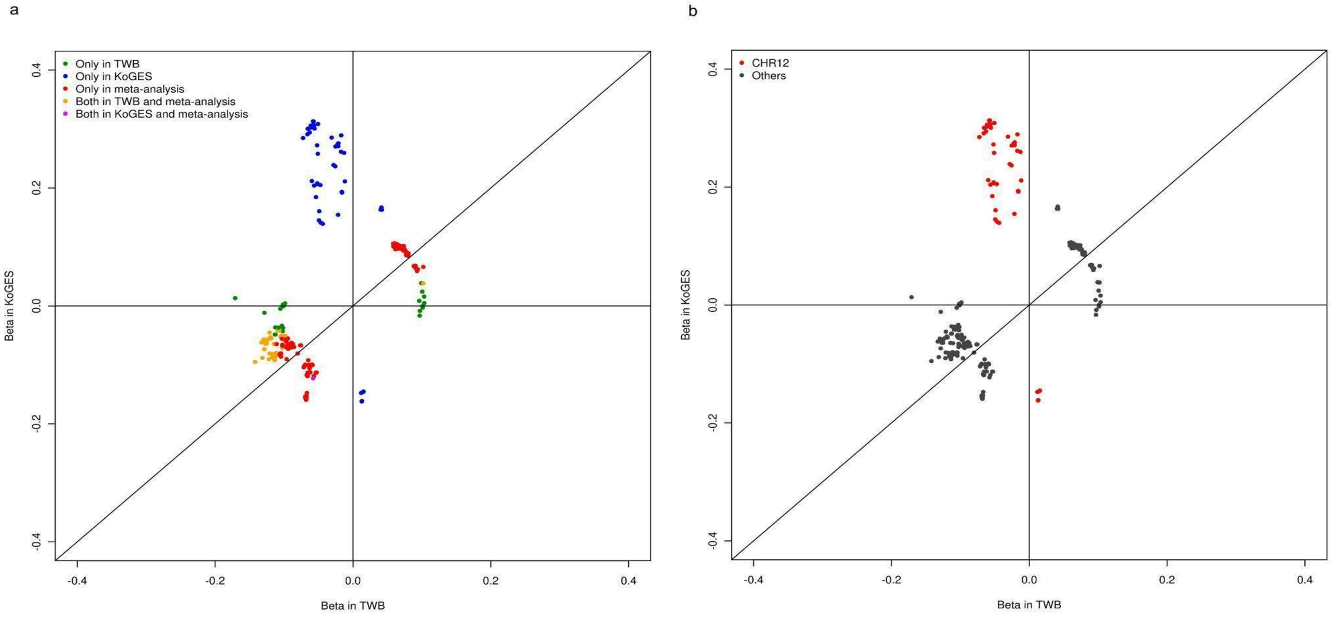

Supplementary Figure 5. Regional association plots

a. Regional association plots for rs10930013. The *x* axis represents the chromosomal position, and the *y* axis represents the $-\log_{10}$(*P*-value) for association of variants with *EduYears* in TWB, KoGES, and genome-wide meta-analysis in East Asian (EAS), respectively. All *P*-values were calculated by a two-sided test. The SNP with the lowest *P*-value was highlighted as a purple diamond in TWB, KoGES, and EAS, respectively. Linkage disequilibrium (LD) estimates of surrounding SNPs with the index SNP (rs10930013 in EAS) are indicated by colour. Red points ($r^2 > 0.8$) show SNPs in high level of LD with the index SNP, and deep blue points ($r^2 < 0.2$) show SNPs in low level of LD with the index SNP.

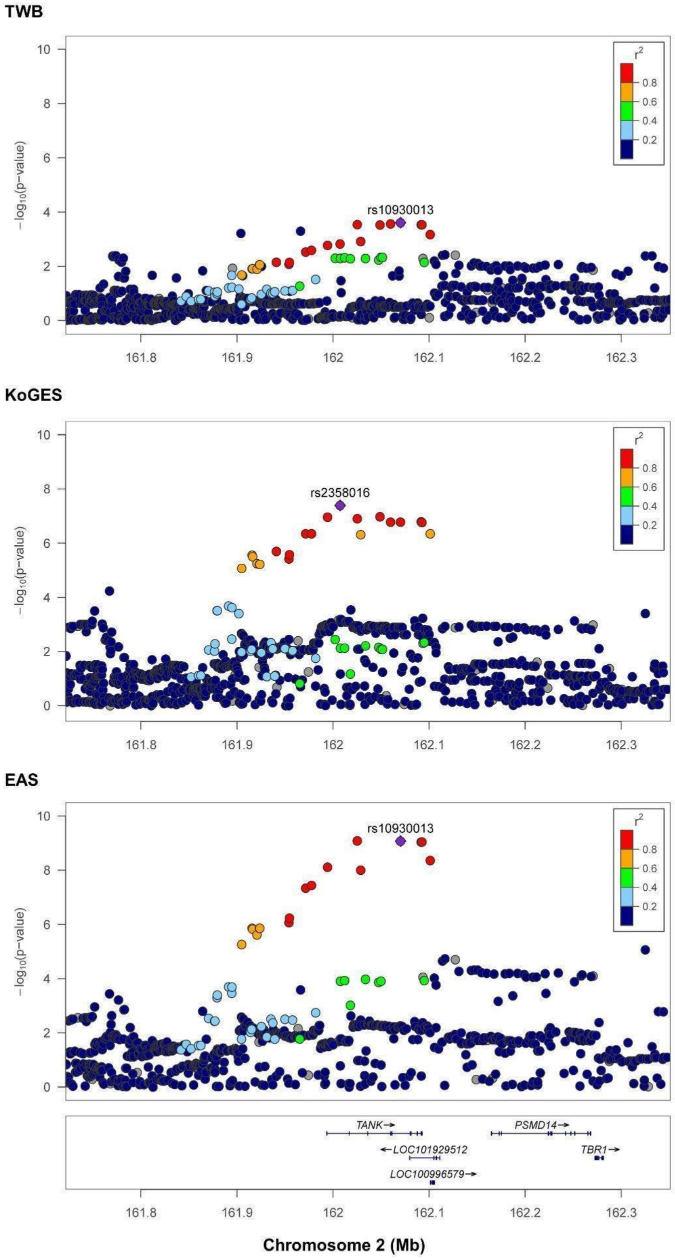

b. Regional association plots for rs2871133. The *x* axis represents the chromosomal position, and the *y* axis represents the $-\log_{10}(P\text{-value})$ for association of variants with *EduYears* in TWB, KoGES, and genome-wide meta-analysis in East Asian (EAS), respectively. All *P*-values were calculated by a two-sided test. The SNP with the lowest *P*-value was highlighted as a purple diamond in TWB, KoGES, and EAS, respectively. Linkage disequilibrium (LD) estimates of surrounding SNPs with the index SNP (rs2871133 in EAS) are indicated by colour. Red points ($r^2 > 0.8$) show SNPs in high level of LD with the index SNP, and deep blue points ($r^2 < 0.2$) show SNPs in low level of LD with the index SNP.

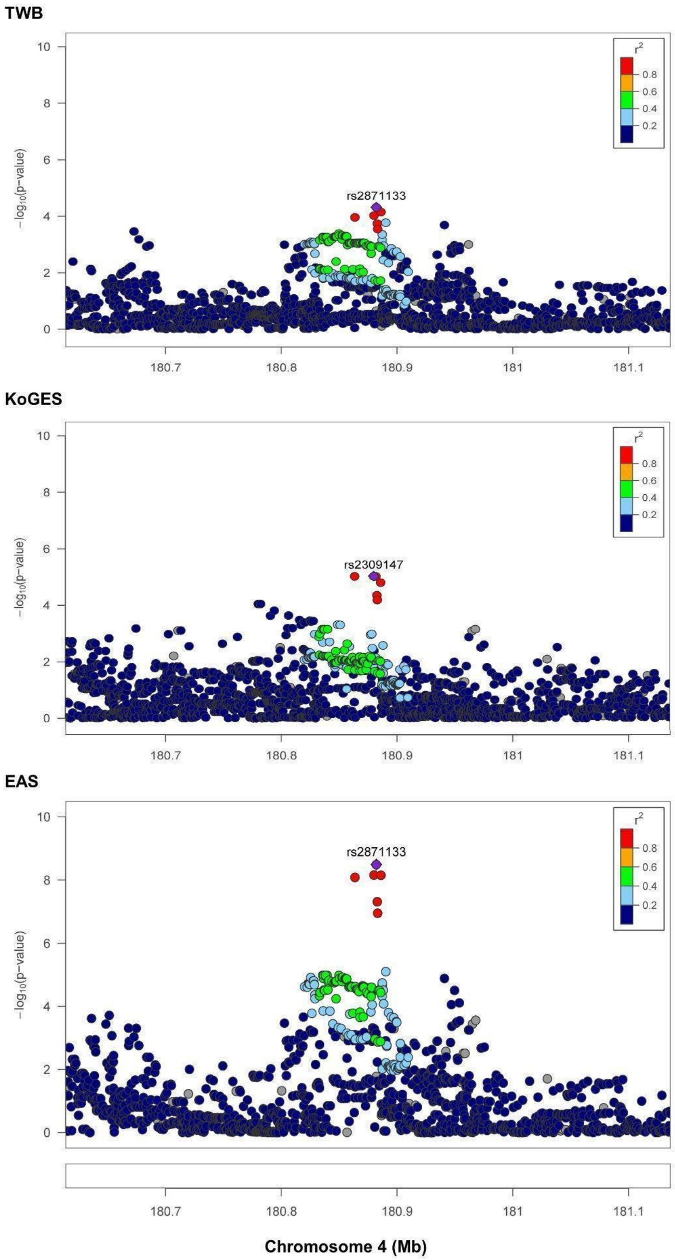

c. Regional association plots for rs255347. The *x* axis represents the chromosomal position, and the *y* axis represents the $-\log_{10}$(*P*-value) for association of variants with *EduYears* in TWB, KoGES, and genome-wide meta-analysis in East Asian (EAS), respectively. All *P*-values were calculated by a two-sided test. The SNP with the lowest *P*-value was highlighted as a purple diamond in TWB, KoGES, and EAS, respectively. Linkage disequilibrium (LD) estimates of surrounding SNPs with the index SNP (rs255347 in EAS) are indicated by colour. Red points (r^2 > 0.8) show SNPs in high level of LD with the index SNP, and deep blue points (r^2 < 0.2) show SNPs in low level of LD with the index SNP.

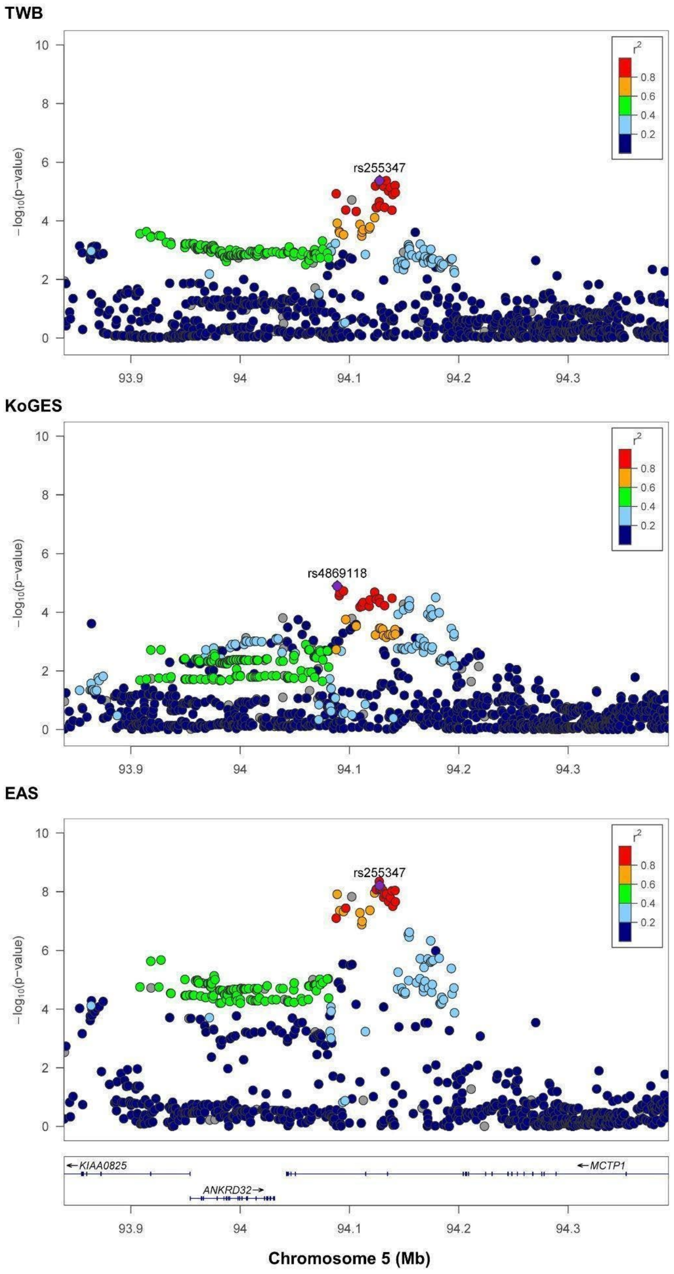

d. Regional association plots for rs7708343. The *x* axis represents the chromosomal position, and the *y* axis represents the $-\log_{10}$(*P*-value) for association of variants with *EduYears* in TWB, KoGES, and genome-wide meta-analysis in East Asian (EAS), respectively. All *P*-values were calculated by a two-sided test. The SNP with the lowest *P*-value was highlighted as a purple diamond in TWB, KoGES, and EAS, respectively. Linkage disequilibrium (LD) estimates of surrounding SNPs with the index SNP (rs7708343 in EAS) are indicated by colour. Red points ($r^2 > 0.8$) show SNPs in high level of LD with the index SNP, and deep blue points ($r^2 < 0.2$) show SNPs in low level of LD with the index SNP.

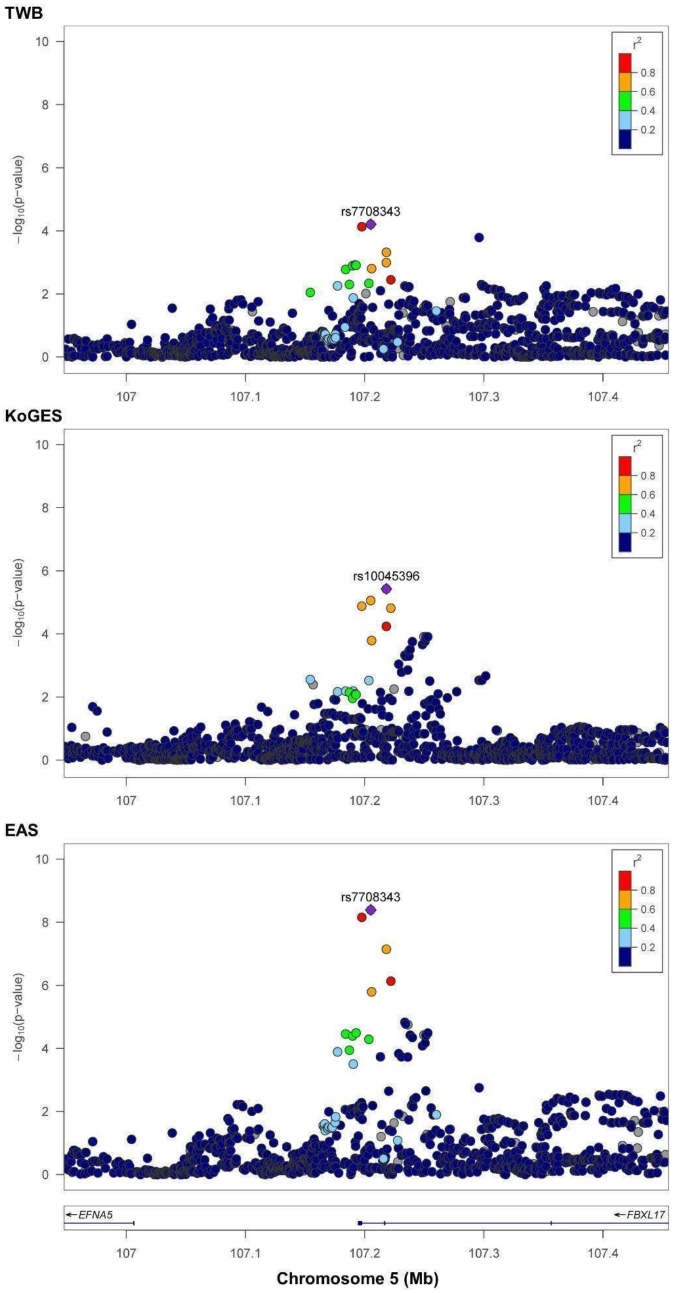

e. Regional association plots for rs16893804. The *x* axis represents the chromosomal position, and the *y* axis represents the $-\log_{10}(P\text{-value})$ for association of variants with *EduYears* in TWB, KoGES, and genome-wide meta-analysis in East Asian (EAS), respectively. All *P*-values were calculated by a two-sided test.The SNP with the lowest *P*-value was highlighted as a purple diamond in TWB, KoGES, and EAS, respectively. Linkage disequilibrium (LD) estimates of surrounding SNPs with the index SNP (rs16893804 in EAS) are indicated by colour. Red points ($r^2 > 0.8$) show SNPs in high level of LD with the index SNP, and deep blue points ($r^2 < 0.2$) show SNPs in low level of LD with the index SNP.

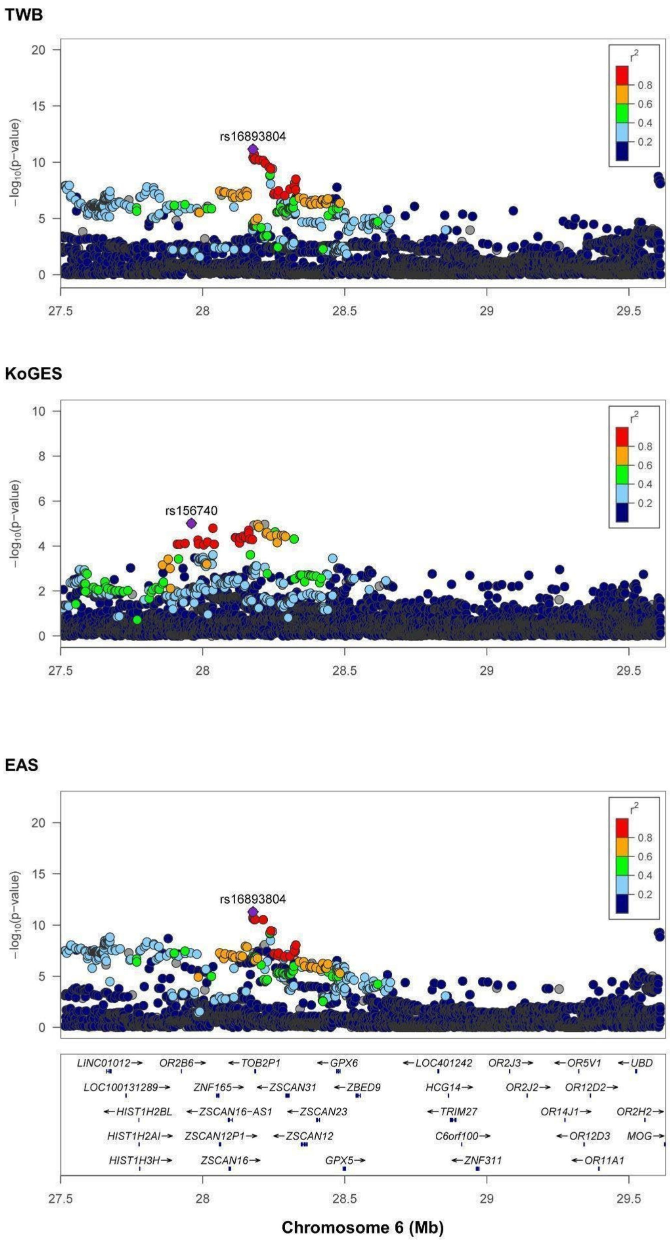

f. Regional association plots for rs11191157. The *x* axis represents the chromosomal position, and the *y* axis represents the $-\log_{10}(P\text{-value})$ for association of variants with *EduYears* in TWB, KoGES, and genome-wide meta-analysis in East Asian (EAS), respectively. All *P*-values were calculated by a two-sided test. The SNP with the lowest *P*-value was highlighted as a purple diamond in TWB, KoGES, and EAS, respectively. Linkage disequilibrium (LD) estimates of surrounding SNPs with the index SNP (rs11191157 in EAS) are indicated by colour. Red points ($r^2 > 0.8$) show SNPs in high level of LD with the index SNP, and deep blue points ($r^2 < 0.2$) show SNPs in low level of LD with the index SNP.

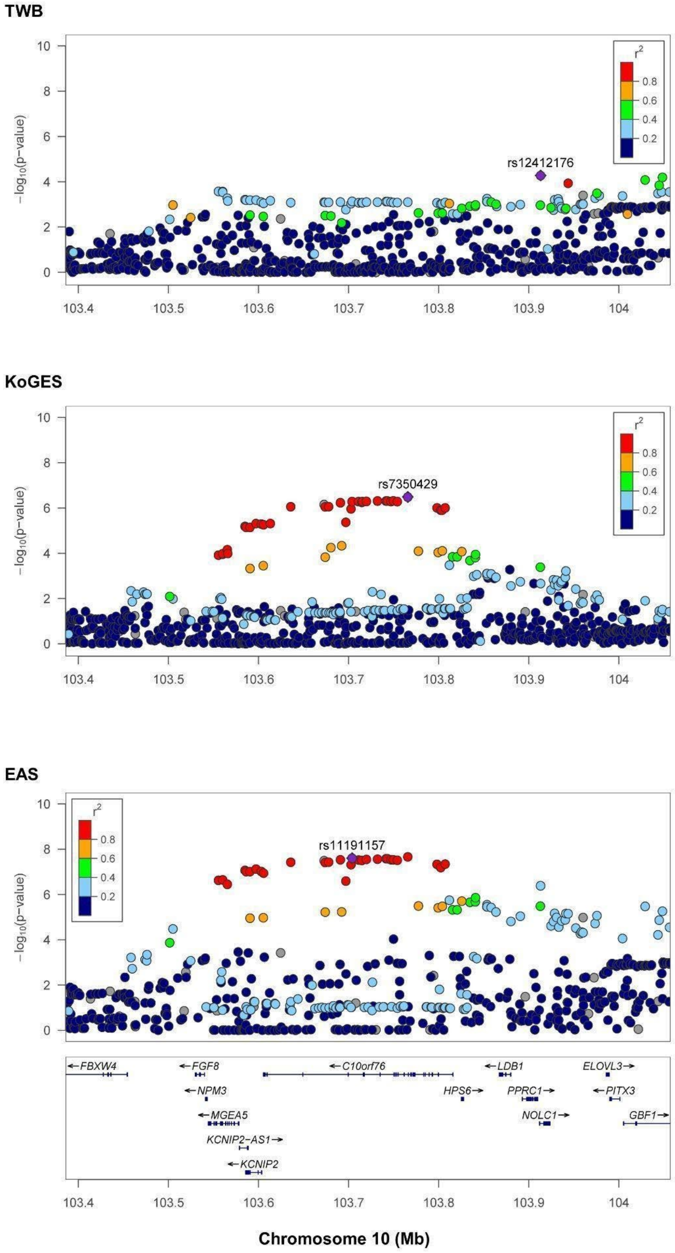

g. Regional association plots for rs12936234. The *x* axis represents the chromosomal position, and the *y* axis represents the $-\log_{10}(P\text{-value})$ for association of variants with *EduYears* in TWB, KoGES, and genome-wide meta-analysis in East Asian (EAS), respectively. All *P*-values were calculated by a two-sided test. The SNP with the lowest *P*-value was highlighted as a purple diamond in TWB, KoGES, and EAS, respectively. Linkage disequilibrium (LD) estimates of surrounding SNPs with the index SNP (rs12936234 in EAS) are indicated by colour. Red points ($r^2 > 0.8$) show SNPs in high level of LD with the index SNP, and deep blue points ($r^2 < 0.2$) show SNPs in low level of LD with the index SNP.

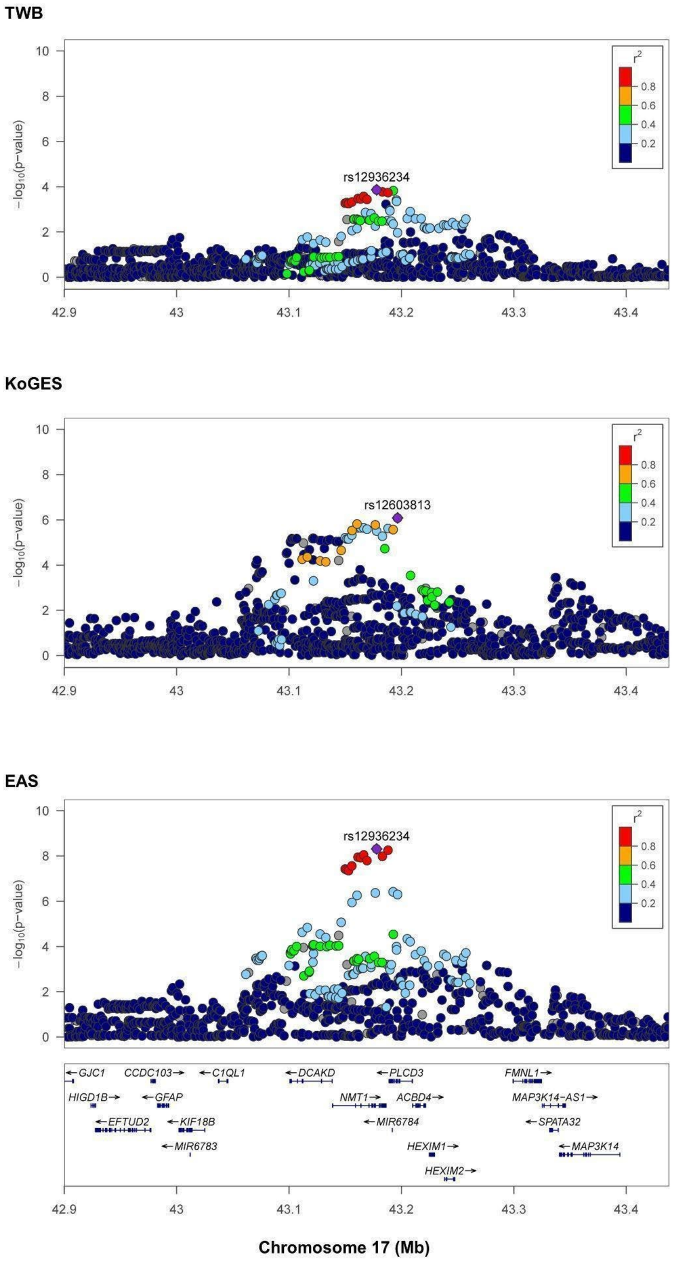

Supplementary Figure 6. The scatter plot of comparison for effect allele frequency between TWB and KoGES. The *x* axis represents effect allele frequency (EAF) in TWB, and the *y* axis represents effect allele frequency in KoGES. Points lie on the diagonal line, highlighted as a red line, indicating that the effect allele frequencies are identical between TWB and KoGES.

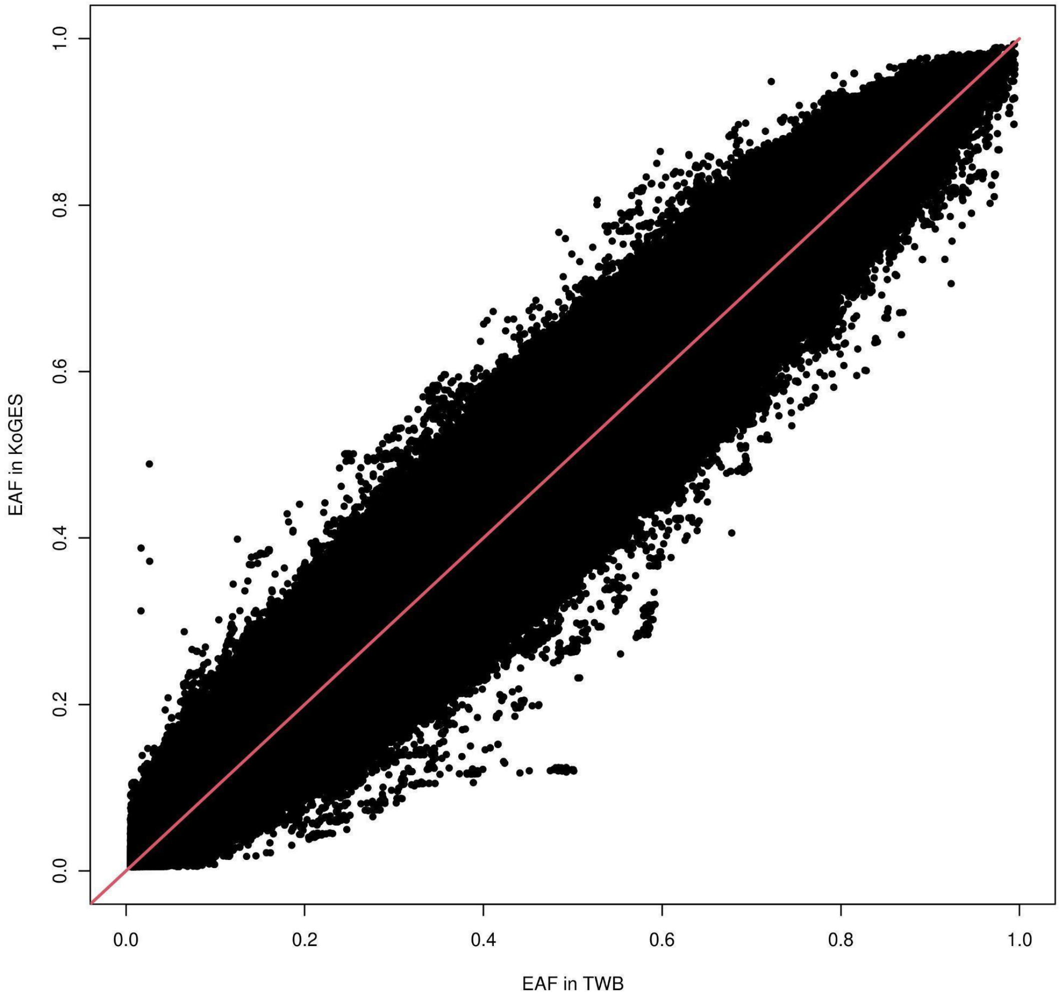

Supplementary Figure 7. a. Manhattan plot of GWAS for *EduYears* in the drinker group of KoGES. b. Manhattan plot of GWAS for *EduYears* in the non-drinker group of KoGES. The x axis represents chromosomal position, and the y axis represents the $-\log_{10}$(P-value) for association of variants with *EduYears*. Reported P-values are two-sided and not corrected for multiple testing. The horizontal red line marks the threshold for genome-wide significance (P-value = 5×10^{-8}), and the horizontal blue line marks the threshold for suggestive genome-wide significance (P-value = 1×10^{-5}).

a

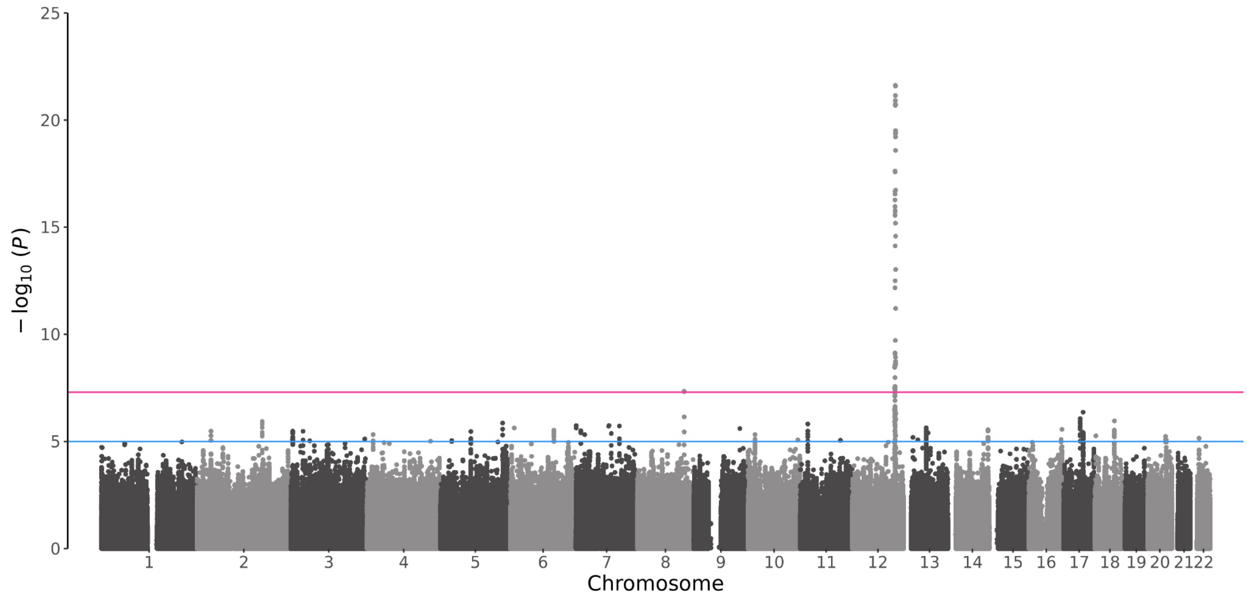

b

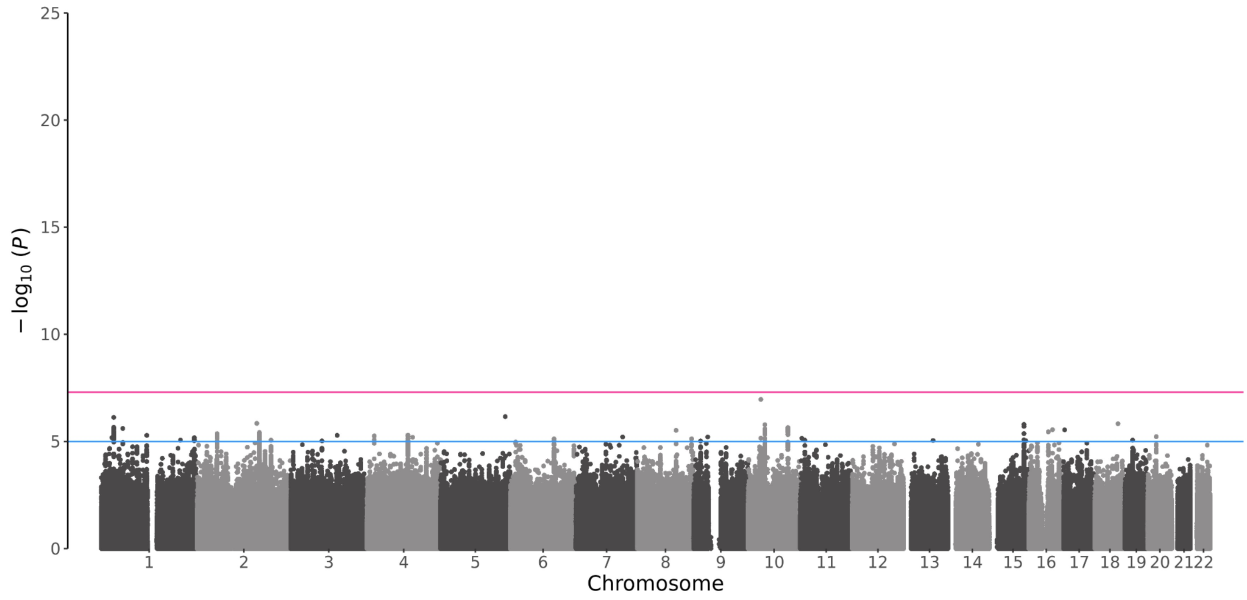

Supplementary Figure 8. SNP-based partitioned heritability in East Asian based on 97 functional genomic annotations. Enrichment estimates for the 97 functional genomic annotations for East Asian *EduYears* GWAS. The *x* axis represents –$\log_{10}$(*P*-value) from a one-sided test, and the *y* axis represents individual functional annotation. Annotations are ordered by *P*-values. The dashed red line indicates FDR-corrected significance at *P*-value < 0.05.

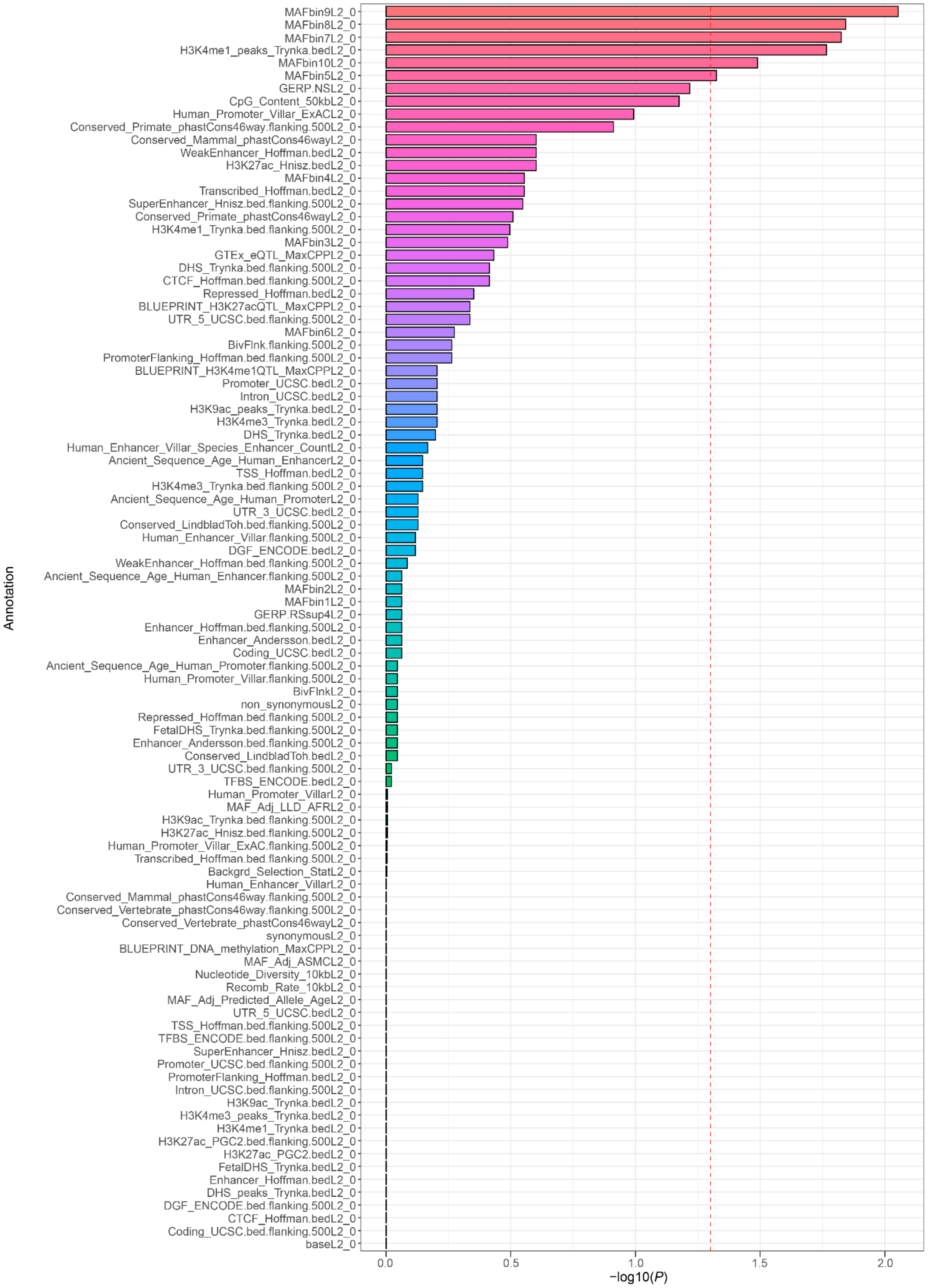

Supplementary Figure 9. Tissue specific gene expression and chromatin enrichment in SNP-based heritability for *EduYears* in East Asian. a. Results of multiple-tissue analysis using gene expression data for East Asian. The *x* axis represents a tissue or cell type, and the *y* axis represents $-\log_{10}$(*P*-value) from a one-sided LDSC-SEG enrichment test. Each point represents a tissue or cell type from either GTEx or Franke laboratory data set. The red dashed line indicates the cut-off for FDR significance at $-\log_{10}$(*P*-value) = 2.44. **b. Results of multiple tissues using chromatin data for East Asian.** The *x* axis represents a tissue or cell type, and the *y* axis represents $-\log_{10}$(*P*-value) from a one-sided LDSC-SEG enrichment test. Each point represents peaks for H3K4me1, H3K27ac, H3K4me3, DNase, or H3K9ac in a tissue or cell type. The red dashed line indicates the cut-off for FDR significance at $-\log_{10}$(*P*-value) = 2.98.

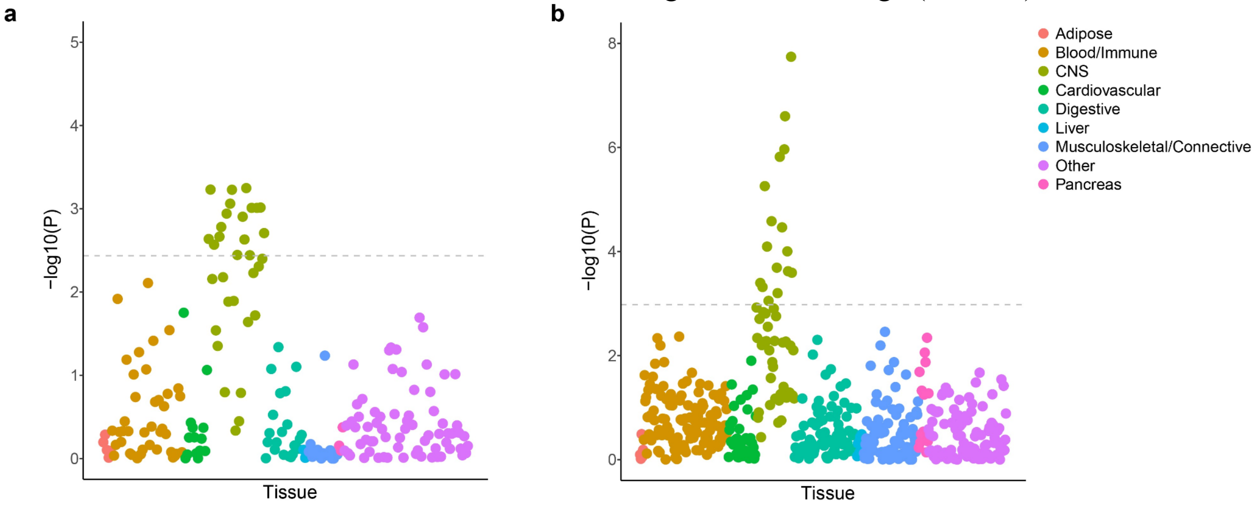

Supplementary Figure 10. Regional association plots for rs2881903 which was identified from EAS-specific cross-population meta-analysis using Multi-Ancestry Meta-Analysis (MAMA). The *x* axis represents the chromosomal position, and the *y* axis represents the –$\log_{10}$(*P*-value) for association of variants with *EduYears* in TWB, KoGES, genome-wide meta-analysis in East Asian (EAS), and genome-wide meta-analysis in European (EUR; Lee *et al.* in 2018), respectively. All *P*-values were calculated by a two-sided test. The SNP with the lowest *P*-value was highlighted as a purple diamond. Linkage disequilibrium (LD) estimates of surrounding SNPs with the index SNP are indicated by colour. Red points ($r^2 > 0.8$) show SNPs in high level of LD with the index SNP, and deep blue points ($r^2 < 0.2$) show SNPs in low level of LD with the index SNP.

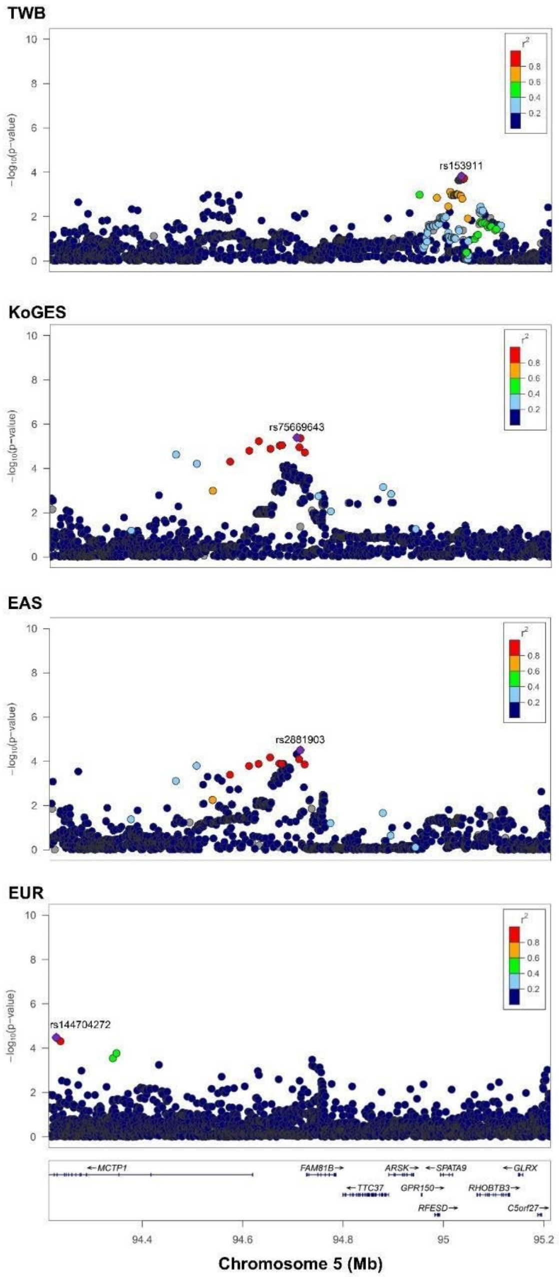

Supplementary Figure 11. Regional association plots for rs16930687 which was identified from EAS-specific cross-population meta-analysis using Multi-Ancestry Meta-Analysis (MAMA). The *x* axis represents the chromosomal position, and the *y* axis represents the $-\log_{10}$(*P*-value) for association of variants with *EduYears* in TWB, KoGES, genome-wide meta-analysis in East Asian (EAS), and genome-wide meta-analysis in European (EUR; Lee *et al*. in 2018), respectively. All *P*-values were calculated by a two-sided test. The SNP with the lowest *P*-value was highlighted as a purple diamond. Linkage disequilibrium (LD) estimates of surrounding SNPs with the index SNP are indicated by colour. Red points ($r^2 > 0.8$) show SNPs in high level of LD with the index SNP, and deep blue points ($r^2 < 0.2$) show SNPs in low level of LD with the index SNP.

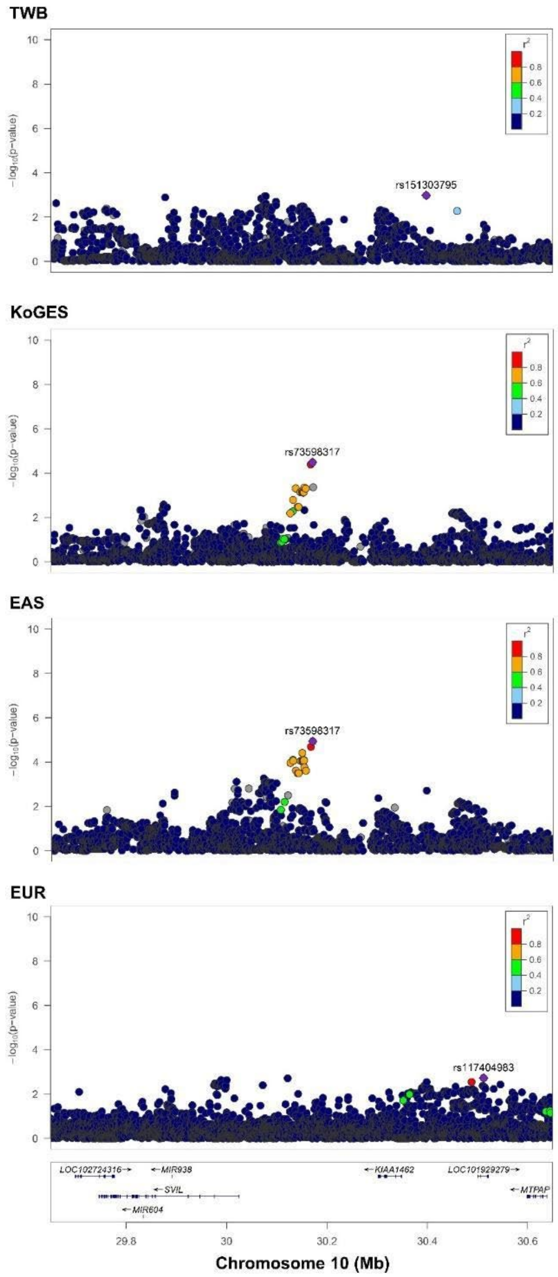

Supplementary Figure 12. Manhattan plot of Multi-Ancestry Meta-Analysis (MAMA) for *EduYears* in European. The x axis represents chromosomal position, and the y axis represents the $-\log_{10}$(P-value) for association of variants with *EduYears*. Reported P-values are two-sided and not corrected for multiple testing. Independent SNPs are highlighted in green. The horizontal pink line marks the threshold for genome-wide significance (P-value = 5×10^{-8}), and the horizontal blue line marks the threshold for suggestive genome-wide significance (P-value = 1×10^{-5}).

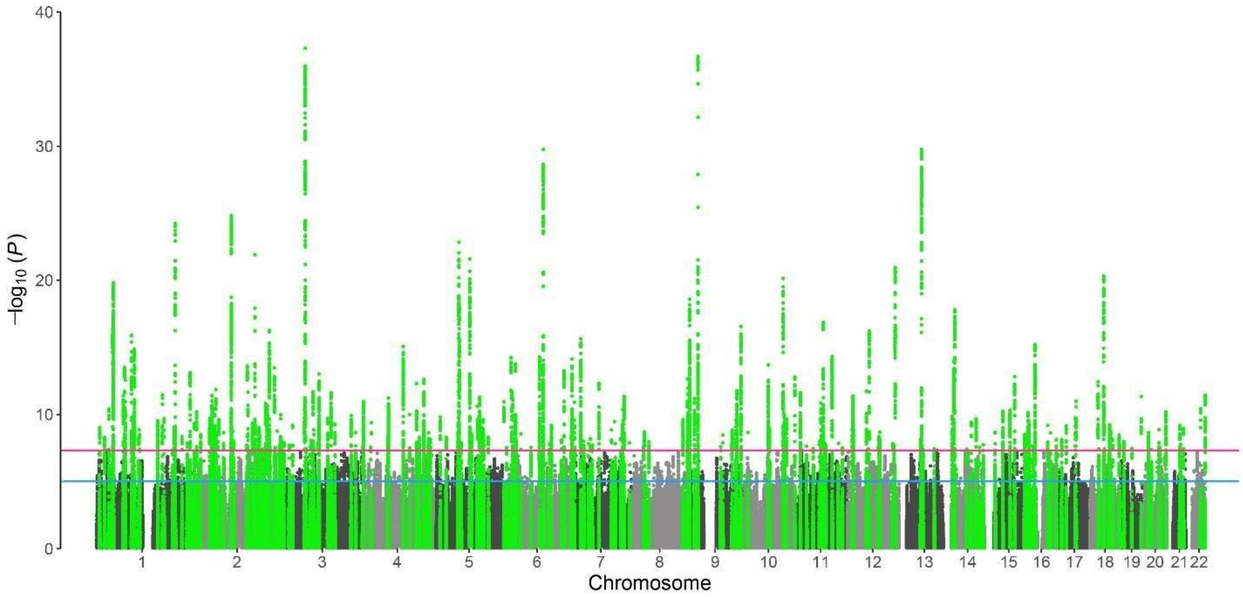

Supplementary Figure 13. Cross- and within-population fine-mapping for *EduYears* in a genome-wide significant locus (rs10930013) in East Asian with *EduYears* GWAS in European by using SuSiEx. a. Regional association plot in East Asian. b. Regional association plot in European. c. Fine-mapping in East Asian. d. Fine-mapping in European. e. Fine-mapping in cross-population. The *x* axis represents the chromosomal position for each panel. The *y* axis represents the $-\log_{10}$(*P*-value) for association of variants with *EduYears* in East Asian and European for panel a and b, respectively. For the meta-analysis of *EduYears* in East Asian, an inverse-variance-weighted fixed-effect model was used. All *P*-values were calculated by a two-sided test and not corrected for multiple testing. In panel a and b, LD estimates of surrounding SNPs with the index SNP are indicated by colour. The y axis represents the posterior inclusion probability (PIP) of the SNPs included in the credible sets identified from fine-mapping in panel c to e. PIP is the probability of being the candidate causal variant for each SNP. We filtered out SNPs with *P*-value > 5×10^{-8}. We marked the value of maximum PIP in each credible set. We identified one credible set from East Asian, European, and cross-population, respectively.

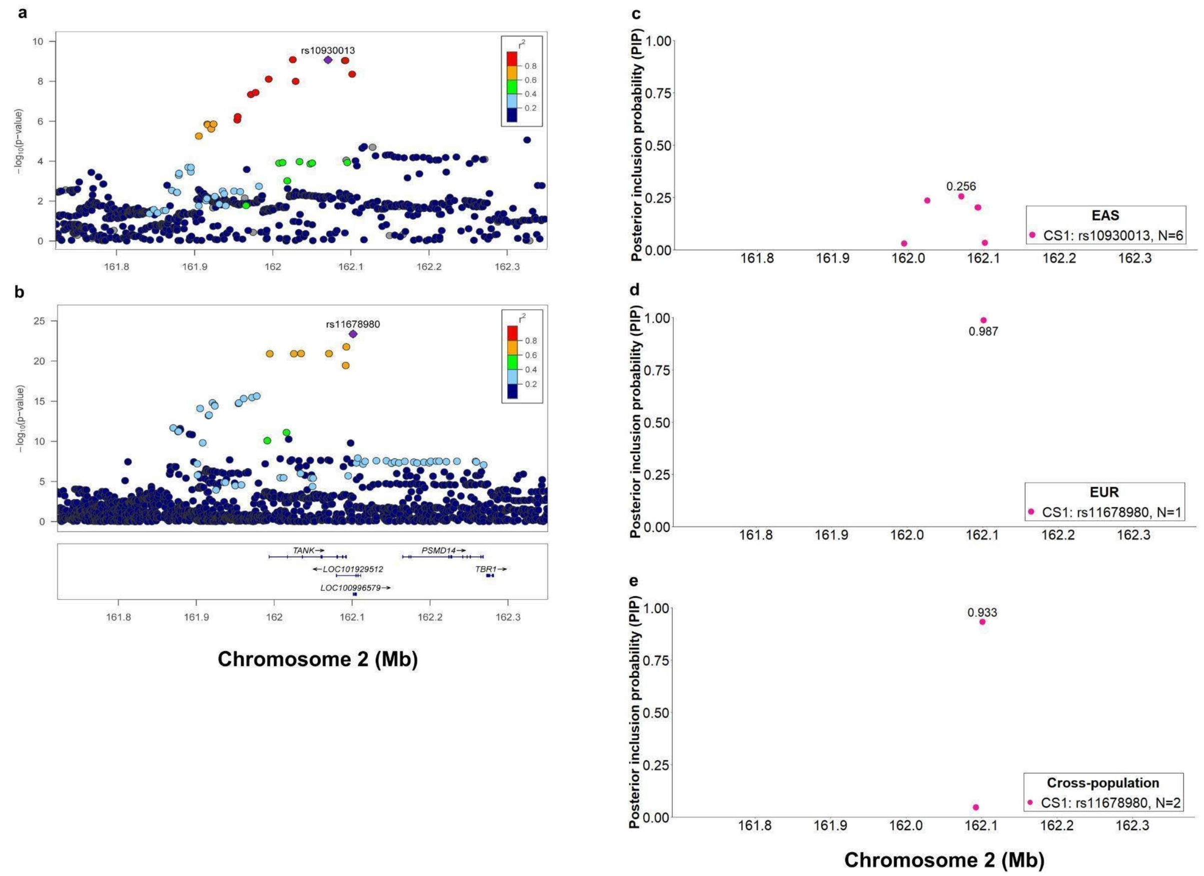

Supplementary Figure 14. Cross- and within-population fine-mapping for *EduYears* in a genome-wide significant locus (rs2871133) in East Asian with *EduYears* GWAS in European by using SuSiEx. a. Regional association plot in East Asian. b. Regional association plot in European. c. Fine-mapping in East Asian. d. Fine-mapping in European. e. Fine-mapping in cross-population. The *x* axis represents the chromosomal position for each panel. The *y* axis represents the $-\log_{10}$(P-value) for association of variants with *EduYears* in East Asian and European for panel a and b, respectively. For the meta-analysis of *EduYears* in East Asian, an inverse-variance-weighted fixed-effect model was used. All P-values were calculated by a two-sided test and not corrected for multiple testing. In panel a and b, LD estimates of surrounding SNPs with the index SNP are indicated by colour. The *y* axis represents the posterior inclusion probability (PIP) of the SNPs included in the credible sets identified from fine-mapping in panel c to e. PIP is the probability of being the candidate causal variant for each SNP. We filtered out SNPs with P-value > 5×10^{-8}. We marked the value of maximum PIP in each credible set. We identified one, zero, and one credible set from East Asian, European, and cross-population, respectively.

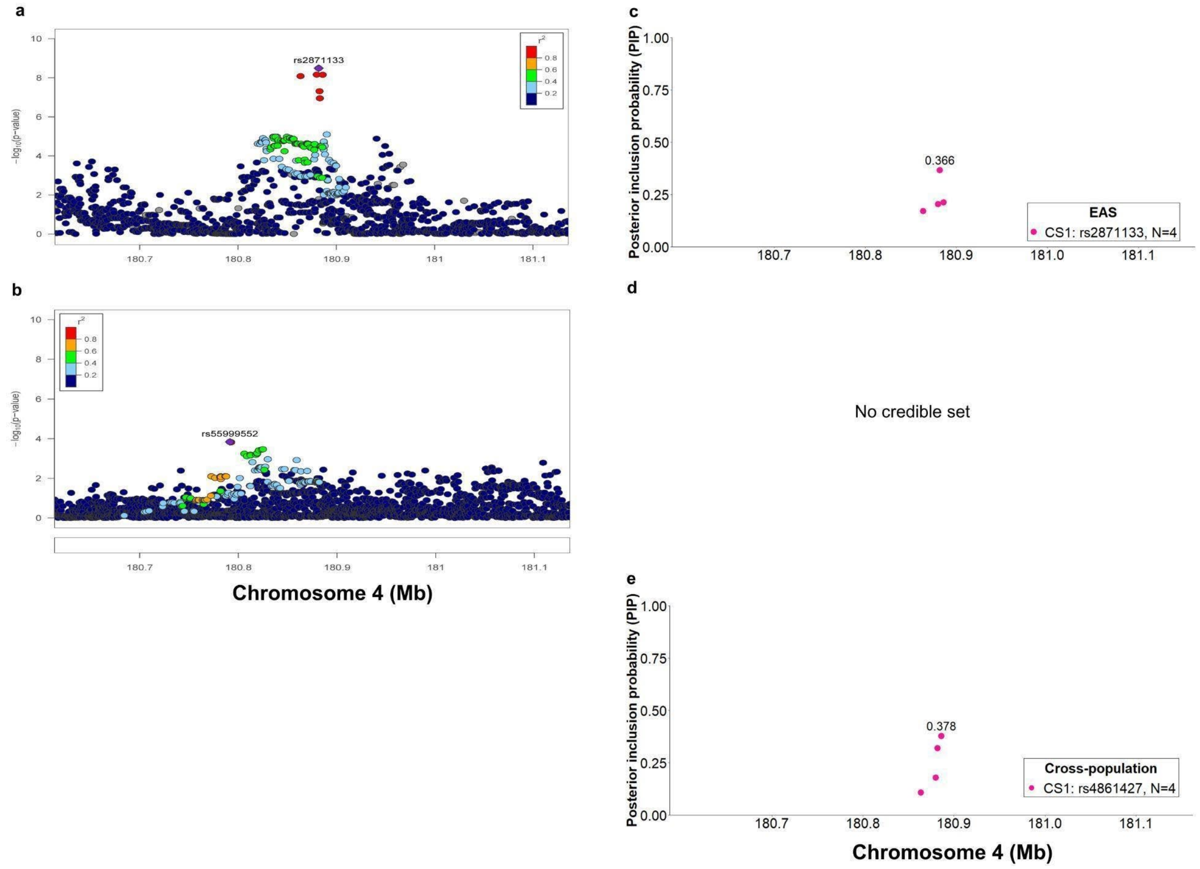

Supplementary Figure 15. Cross- and within-population fine-mapping for *EduYears* in a genome-wide significant locus (rs255347) in East Asian with *EduYears* GWAS in European by using SuSiEx. a. Regional association plot in East Asian. b. Regional association plot in European. c. Fine-mapping in East Asian. d. Fine-mapping in European. e. Fine-mapping in cross-population. The *x* axis represents the chromosomal position for each panel. The *y* axis represents the $-\log_{10}(P\text{-value})$ for association of variants with *EduYears* in East Asian and European for panel a and b, respectively. For the meta-analysis of *EduYears* in East Asian, an inverse-variance-weighted fixed-effect model was used. All *P*-values were calculated by a two-sided test and not corrected for multiple testing. In panel a and b, LD estimates of surrounding SNPs with the index SNP are indicated by colour. The *y* axis represents the posterior inclusion probability (PIP) of the SNPs included in the credible sets identified from fine-mapping in panel c to e. PIP is the probability of being the candidate causal variant for each SNP. We filtered out SNPs with *P*-value > 5×10^{-8}. We marked the value of maximum PIP in each credible set. We identified one, zero, and one credible set from East Asian, European, and cross-population, respectively.

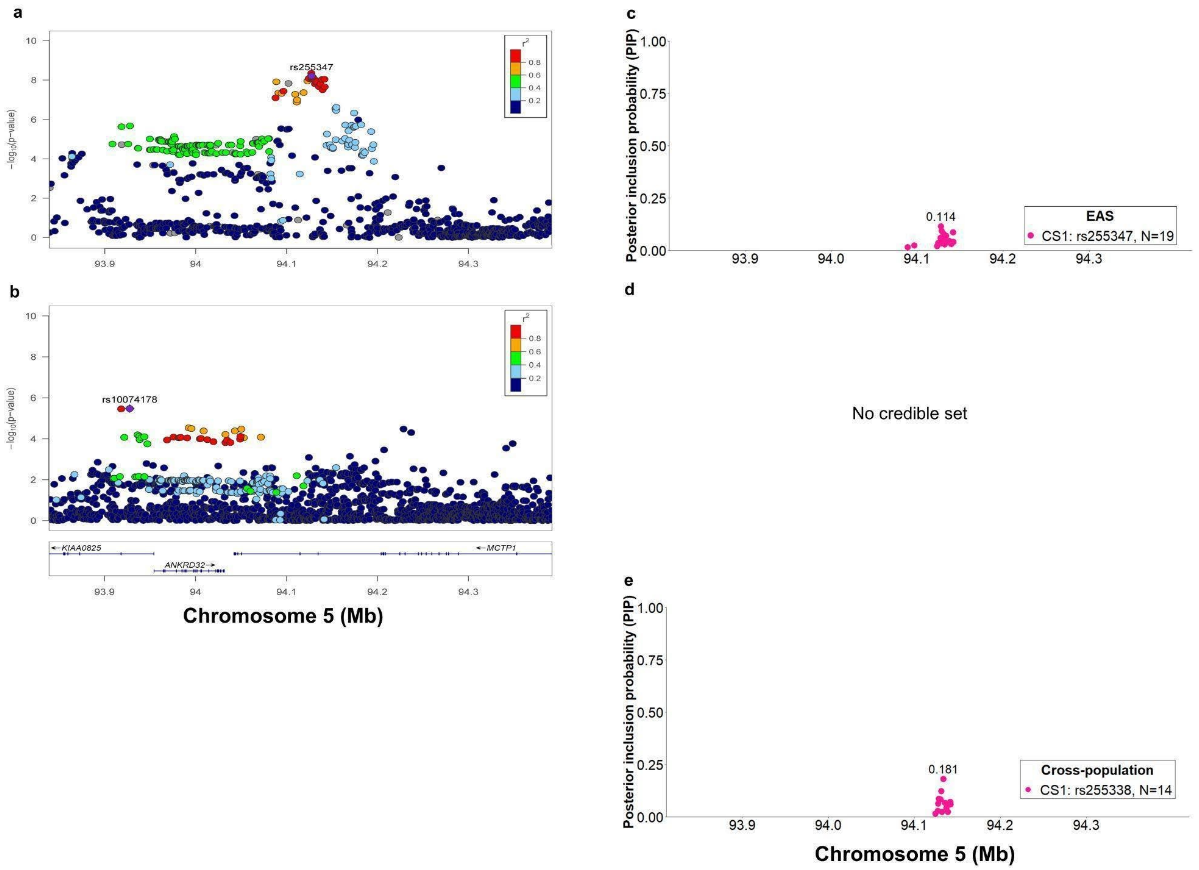

Supplementary Figure 16. Cross- and within-population fine-mapping for *EduYears* in a genome-wide significant locus (rs7708343) in East Asian with *EduYears* GWAS in European by using SuSiEx. a. Regional association plot in East Asian. b. Regional association plot in European. c. Fine-mapping in East Asian. d. Fine-mapping in European. e. Fine-mapping in cross-population. The *x* axis represents the chromosomal position for each panel. The *y* axis represents the $-\log_{10}$(*P*-value) for association of variants with *EduYears* in East Asian and European for panel a and b, respectively. For the meta-analysis of *EduYears* in East Asian, an inverse-variance-weighted fixed-effect model was used. All *P*-values were calculated by a two-sided test and not corrected for multiple testing. In panel a and b, LD estimates of surrounding SNPs with the index SNP are indicated by colour. The *y* axis represents the posterior inclusion probability (PIP) of the SNPs included in the credible sets identified from fine-mapping in panel c to e. PIP is the probability of being the candidate causal variant for each SNP. We filtered out SNPs with *P*-value > 5×10^{-8}. We marked the value of maximum PIP in each credible set, and we used different colours to distinguish each credible set. For example, there are one (pink points), two (blue points and green points), and two (pink points and green points) credible sets identified from East Asian, European, and cross-population, respectively.

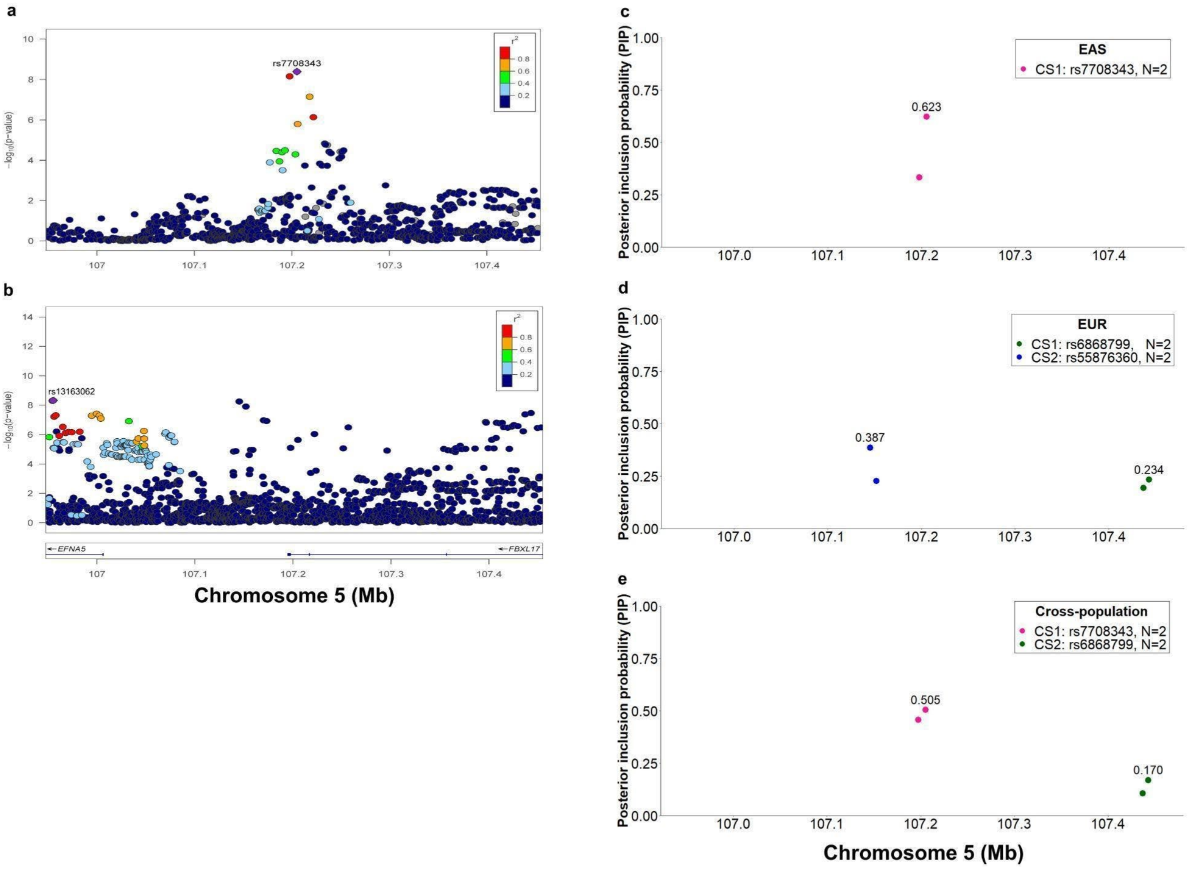

Supplementary Figure 17. Cross- and within-population fine-mapping for *EduYears* in a genome-wide significant locus (rs16893804) in East Asian with *EduYears* GWAS in European by using SuSiEx. a. Regional association plot in East Asian. b. Regional association plot in European. c. Fine-mapping in East Asian. d. Fine-mapping in European. e. Fine-mapping in cross-population. The *x* axis represents the chromosomal position for each panel. The *y* axis represents the $-\log_{10}$(*P*-value) for association of variants with *EduYears* in East Asian and European for panel a and b, respectively. For the meta-analysis of *EduYears* in East Asian, an inverse-variance-weighted fixed-effect model was used. All *P*-values were calculated by a two-sided test and not corrected for multiple testing. In panel a and b, LD estimates of surrounding SNPs with the index SNP are indicated by colour. The *y* axis represents the posterior inclusion probability (PIP) of the SNPs included in the credible sets identified from fine-mapping in panel c to e. PIP is the probability of being the candidate causal variant for each SNP. We filtered out SNPs with *P*-value > 5×10^{-8}. We marked the value of maximum PIP in each credible set, and we used different colours to distinguish each credible set. For example, there are two (blue points and green points), one (pink points), and two (pink points and green points) credible sets identified from East Asian, European, and cross-population, respectively.

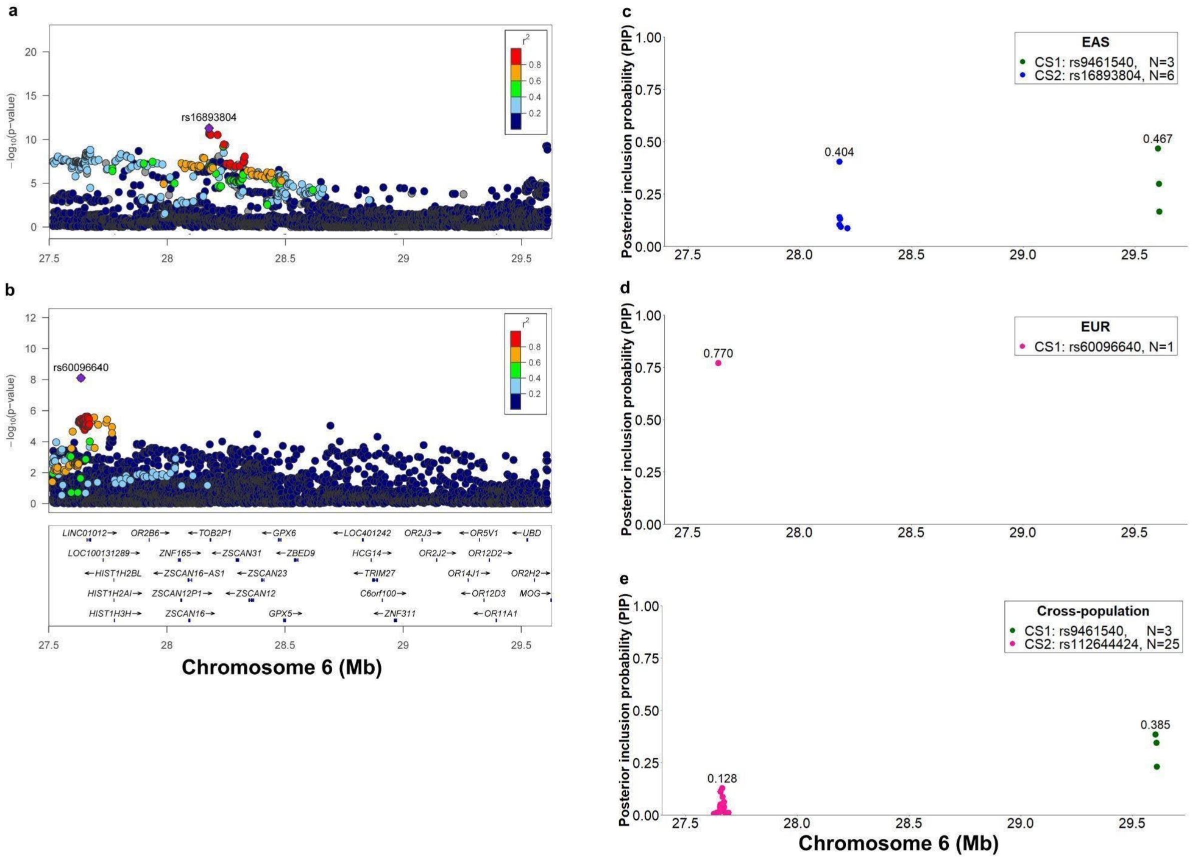

Supplementary Figure 18. Cross- and within-population fine-mapping for *EduYears* in a genome-wide significant locus (rs11191157) in East Asian with *EduYears* GWAS in European by using SuSiEx. a. Regional association plot in East Asian. b. Regional association plot in European. c. Fine-mapping in East Asian. d. Fine-mapping in European. e. Fine-mapping in cross-population. The *x* axis represents the chromosomal position for each panel. The *y* axis represents the $-\log_{10}(P\text{-value})$ for association of variants with *EduYears* in East Asian and European for panel a and b, respectively. For the meta-analysis of *EduYears* in East Asian, an inverse-variance-weighted fixed-effect model was used. All *P*-values were calculated by a two-sided test and not corrected for multiple testing. In panel a and b, LD estimates of surrounding SNPs with the index SNP are indicated by colour. The *y* axis represents the posterior inclusion probability (PIP) of the SNPs included in the credible sets identified from fine-mapping in panel c to e. PIP is the probability of being the candidate causal variant for each SNP. We filtered out SNPs with *P*-value > 5×10^{-8}. We marked the value of maximum PIP in each credible set, and we used different colours to distinguish each credible set. For example, there are one (pink points), two (pink points and blue points), and two (pink points and blue points) credible sets identified from East Asian, European, and cross-population, respectively.

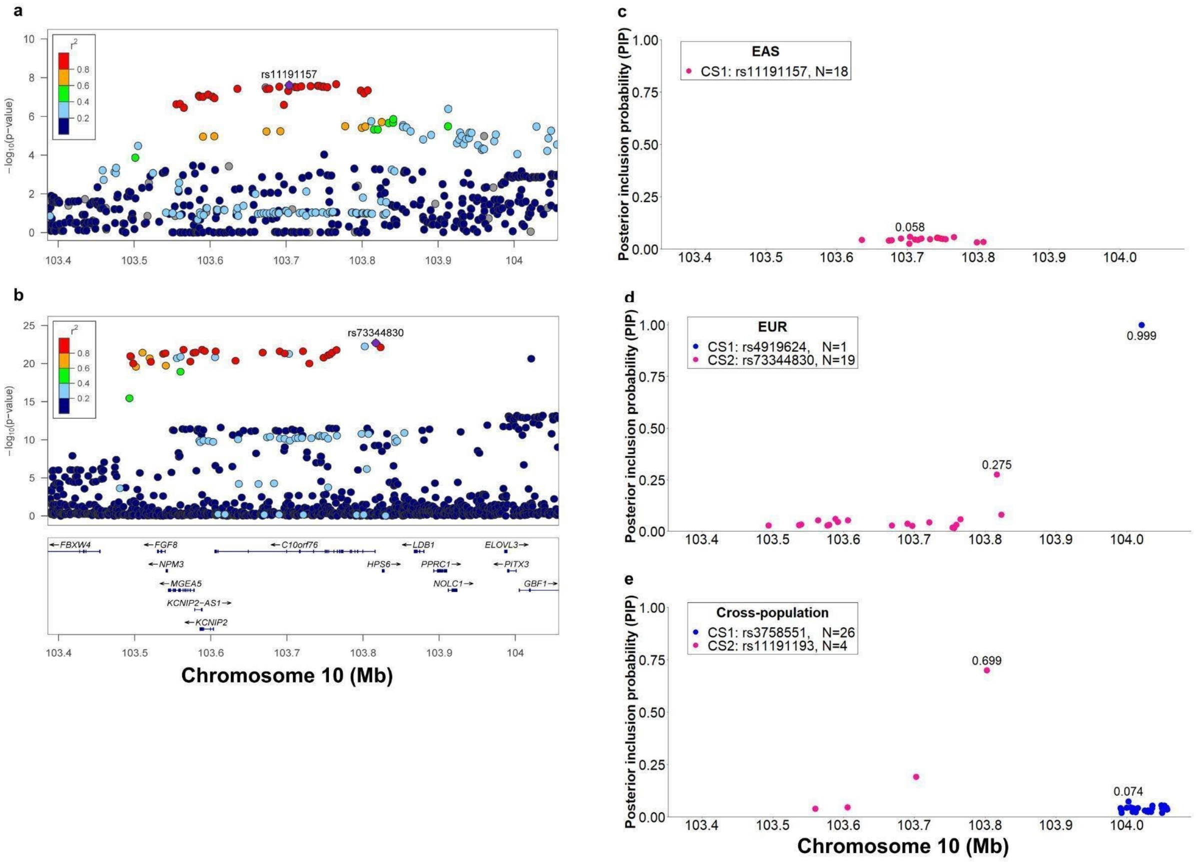

Supplementary Figure 19. Scatter plot of the consistency between the GWAS summary statistics and the LD matrix from the reference panel

a. **Scatter plot of the consistency between the GWAS summary statistics and the LD matrix from the reference panel for SNPs on chromosome 2:161,721,597-162,351,261.** The *x* axis represents the expected z scores (LD matrix from the reference panel), and the y axis represents the observed z scores (the GWAS summary statistics). Points lie along the diagonal indicating consistency between the observed value and the expected value. "s" gives information about the consistency of the LD. A larger "s" metric implies a strong inconsistency between GWAS summary statistics and the LD matrix from the reference panel.

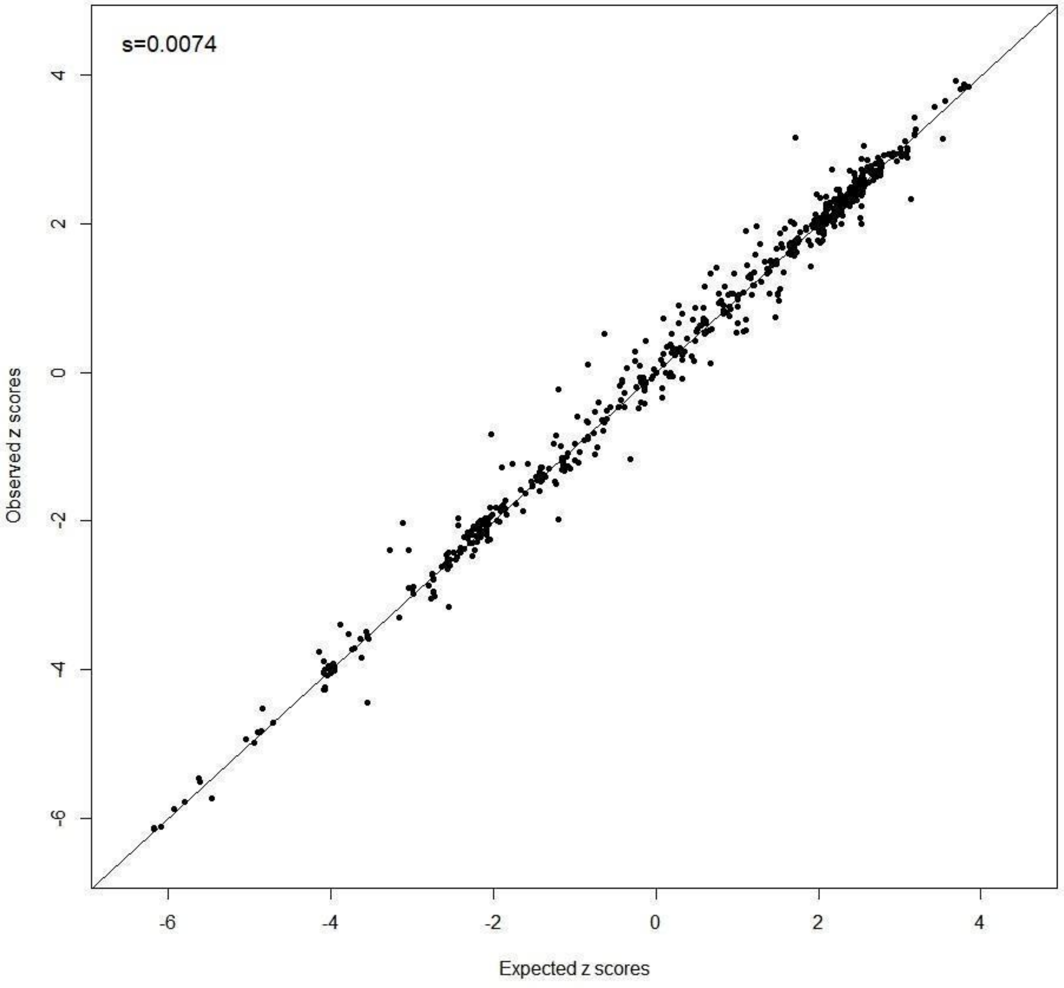

b. **Scatter plot of the consistency between the GWAS summary statistics and the LD matrix from the reference panel for SNPs on chromosome 4:180,613,679-181,136,169.** The *x* axis represents the expected z scores (LD matrix from the reference panel), and the *y* axis represents the observed z scores (the GWAS summary statistics). Points lie along the diagonal indicating consistency between the observed value and the expected value. "s" gives information about the consistency of the LD. A larger "s" metric implies a strong inconsistency between GWAS summary statistics and the LD matrix from the reference panel.

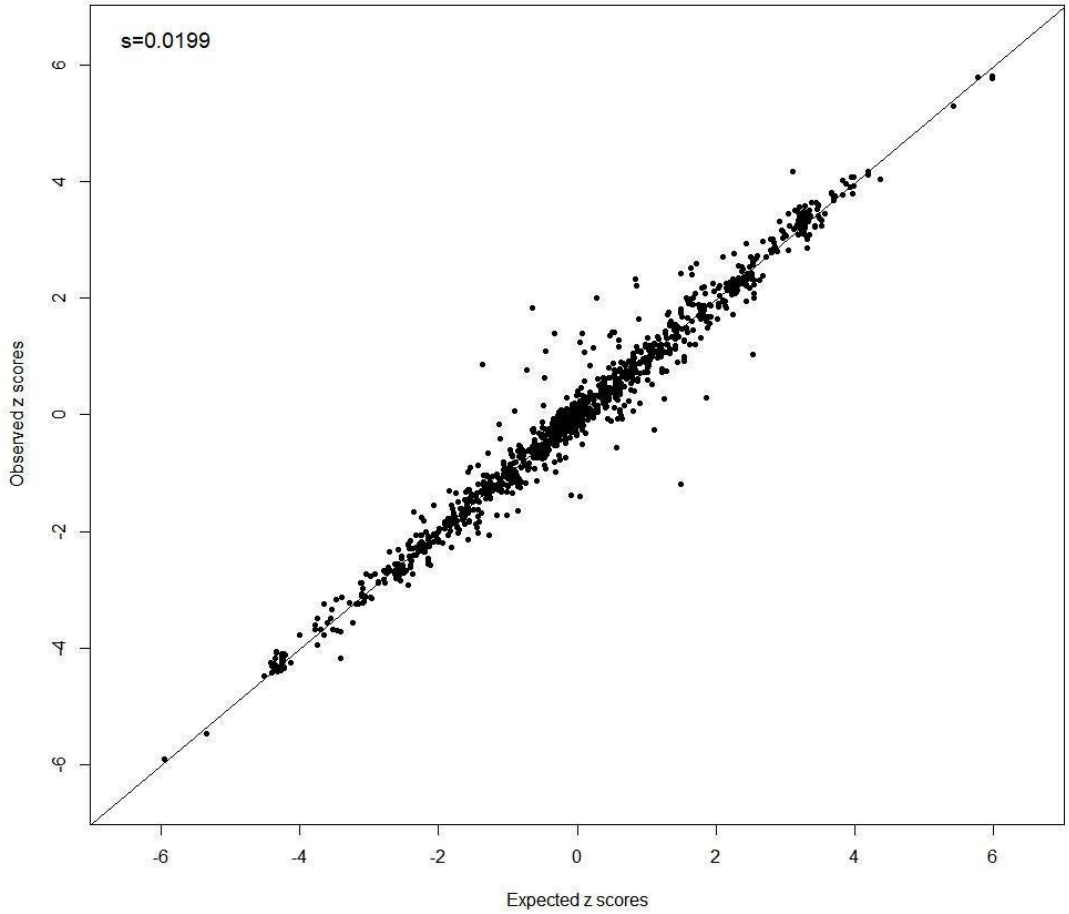

c. Scatter plot of the consistency between the GWAS summary statistics and the LD matrix from the reference panel for SNPs on chromosome 5:93,838,858-94,392,030. The *x* axis represents the expected z scores (LD matrix from the reference panel), and the *y* axis represents the observed z scores (the GWAS summary statistics). Points lie along the diagonal indicating consistency between the observed value and the expected value. "s" gives information about the consistency of the LD. A larger "s" metric implies a strong inconsistency between GWAS summary statistics and the LD matrix from the reference panel.

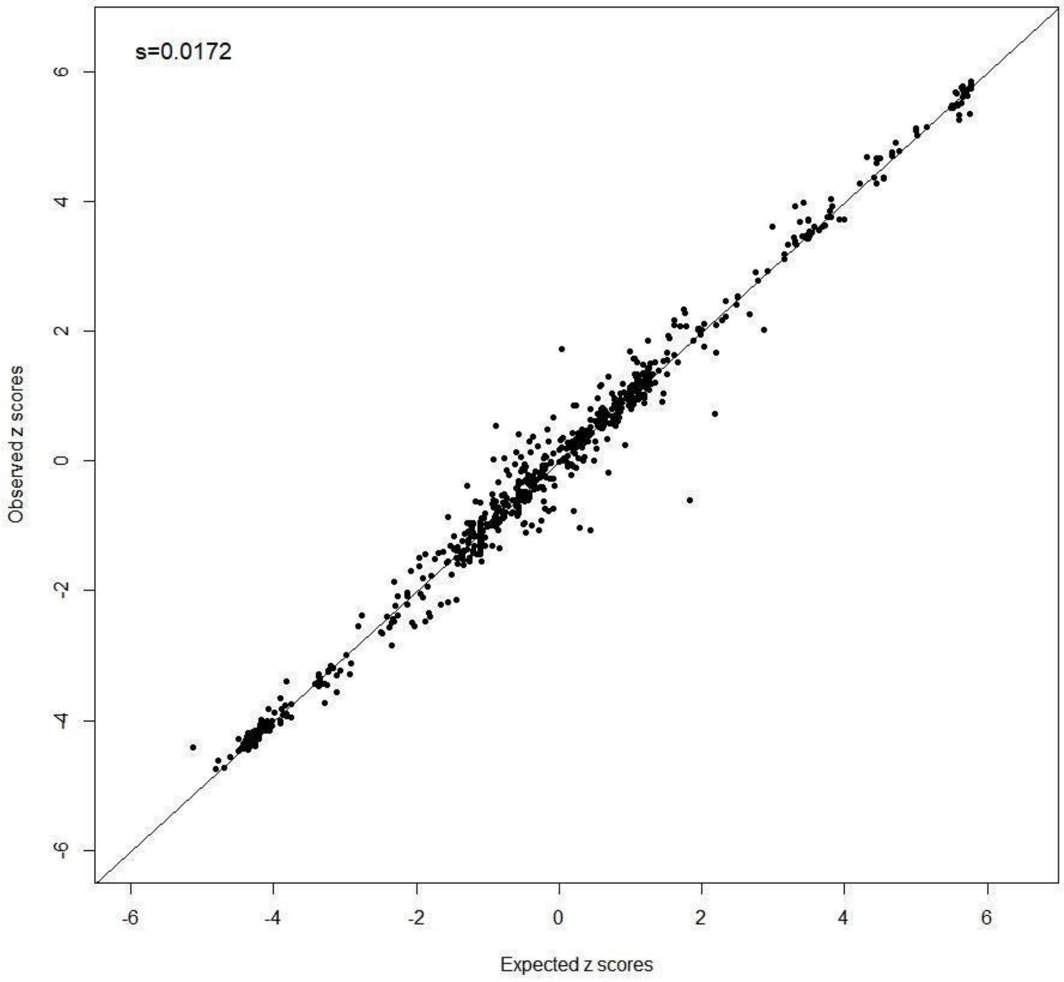

d. Scatter plot of the consistency between the GWAS summary statistics and the LD matrix from the reference panel for SNPs on chromosome 5:106,947,725-107,455,182. The *x* axis represents the expected z scores (LD matrix from the reference panel), and the *y* axis represents the observed z scores (the GWAS summary statistics). Points lie along the diagonal indicating consistency between the observed value and the expected value. "s" gives information about the consistency of the LD. A larger "s" metric implies a strong inconsistency between GWAS summary statistics and the LD matrix from the reference panel.

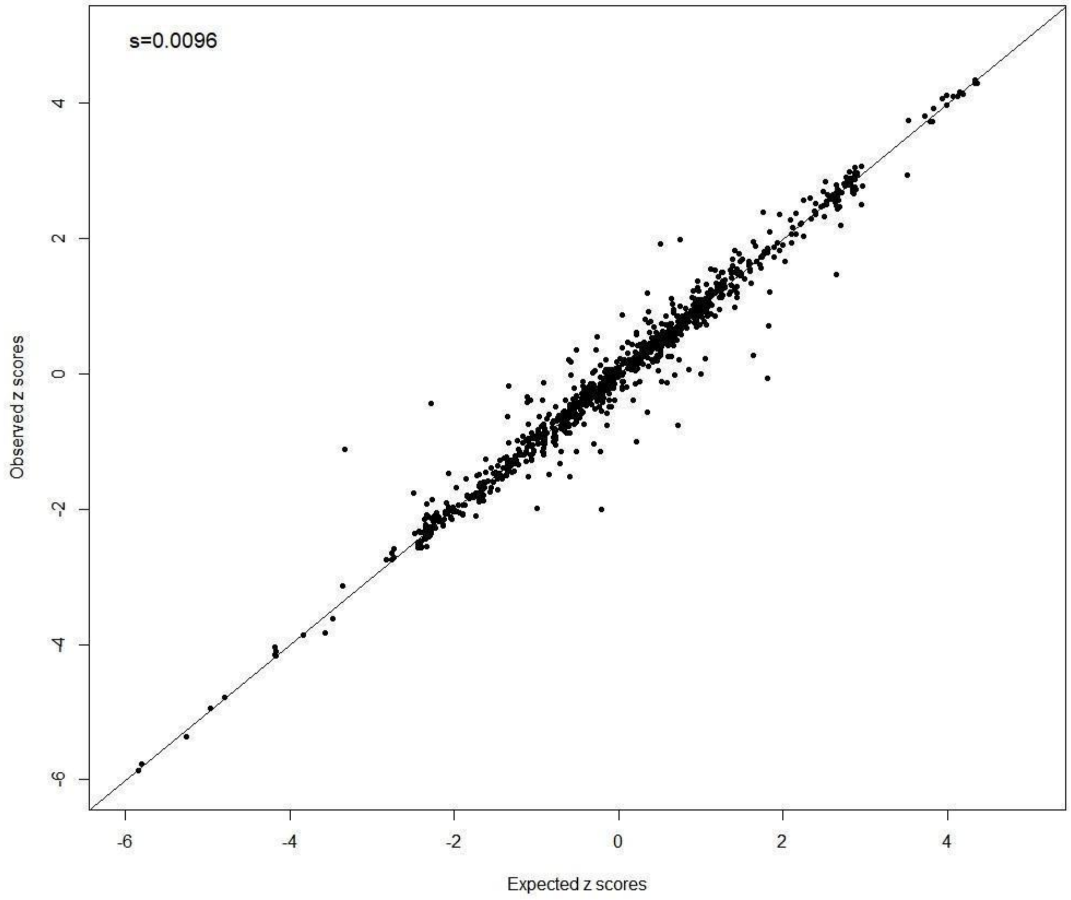

e. Scatter plot of the consistency between the GWAS summary statistics and the LD matrix from the reference panel for SNPs on chromosome 6:27,515,505-29,611,229. The *x* axis represents the expected z scores (LD matrix from the reference panel), and the *y* axis represents the observed z scores (the GWAS summary statistics). Points lie along the diagonal indicating consistency between the observed value and the expected value. "s" gives information about the consistency of the LD. A larger "s" metric implies a strong inconsistency between GWAS summary statistics and the LD matrix from the reference panel.

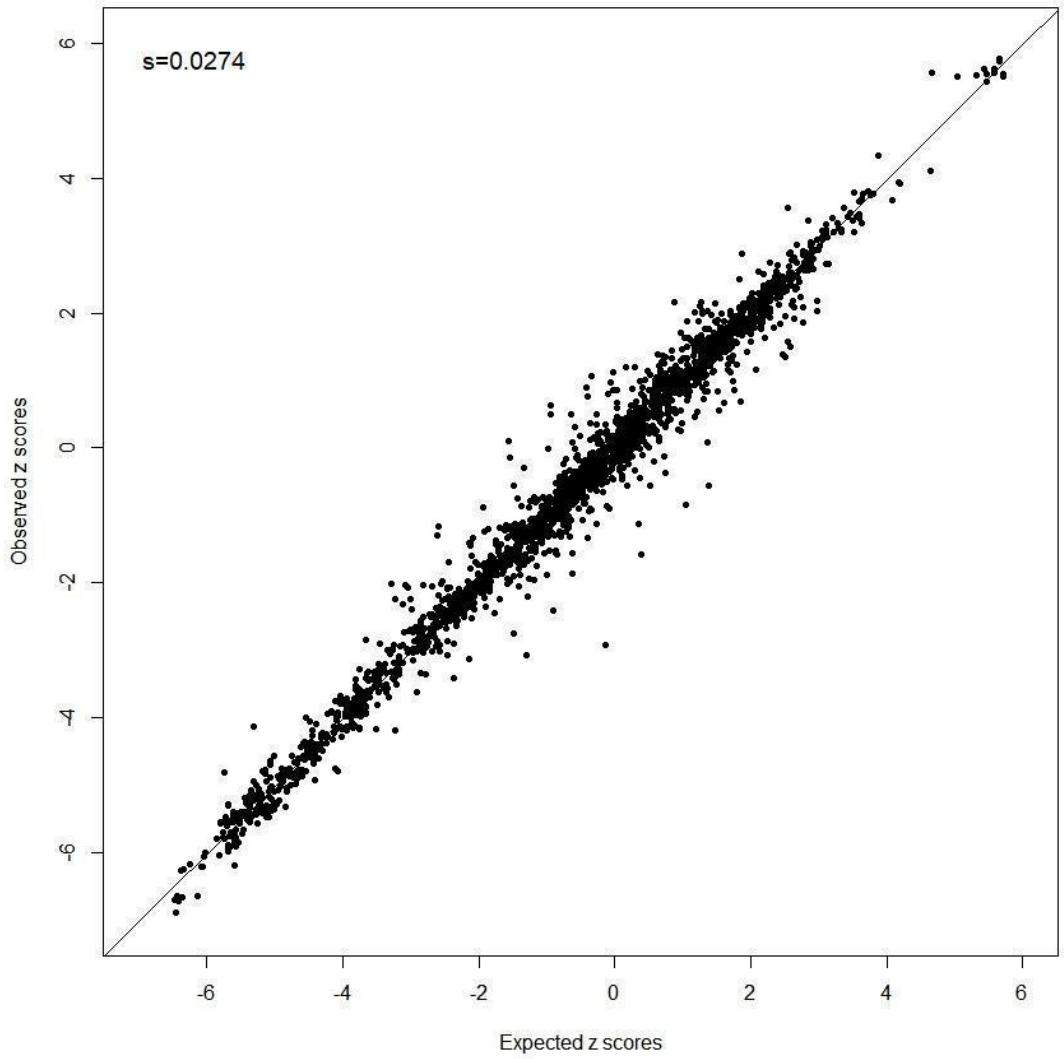

f. Scatter plot of the consistency between the GWAS summary statistics and the LD matrix from the reference panel for SNPs on chromosome 10:103,385,878-104,057,295. The *x* axis represents the expected z scores (LD matrix from the reference panel), and the *y* axis represents the observed z scores (the GWAS summary statistics). Points lie along the diagonal indicating consistency between the observed value and the expected value. "s" gives information about the consistency of the LD. A larger "s" metric implies a strong inconsistency between GWAS summary statistics and the LD matrix from the reference panel.

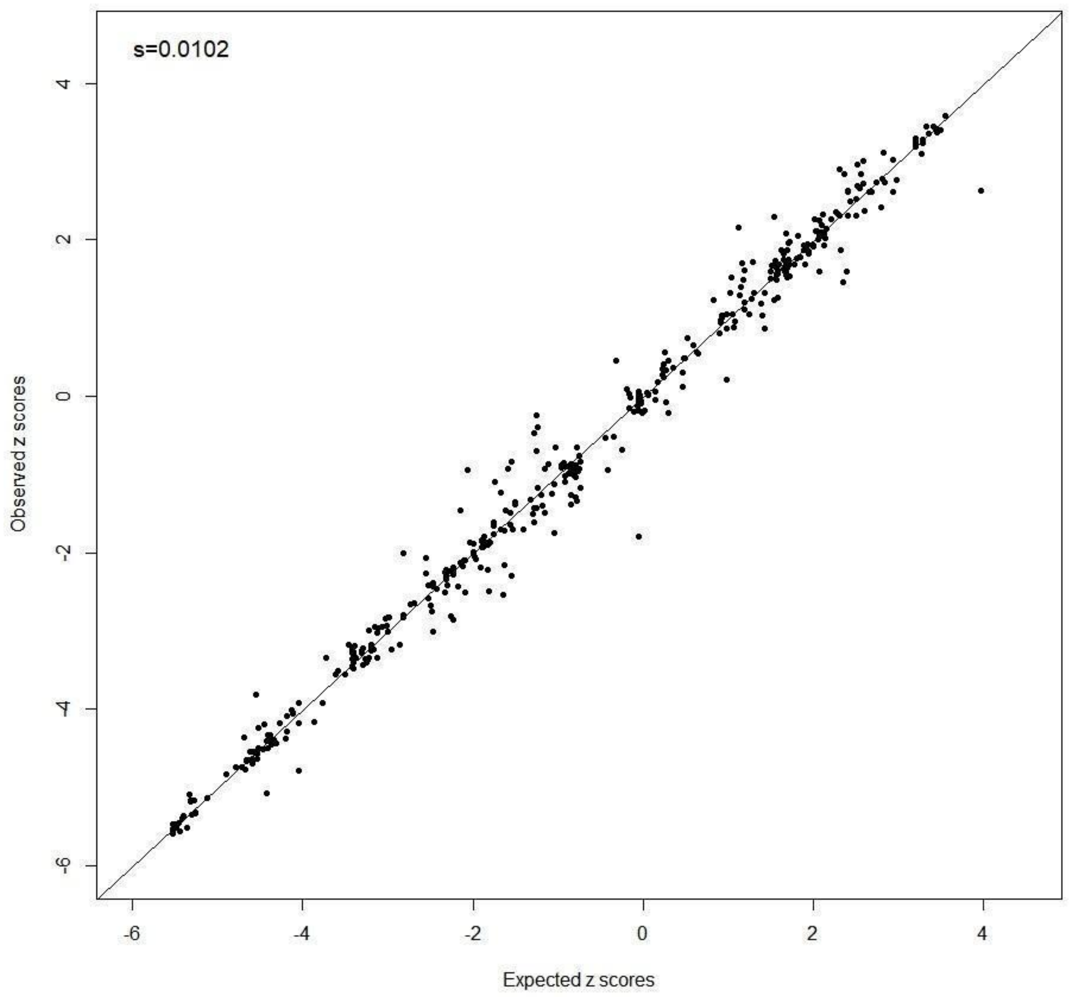

g. Scatter plot of the consistency between the GWAS summary statistics and the LD matrix from the reference panel for SNPs on chromosome 17:42,899,988-43,438,117. The *x* axis represents the expected z scores (LD matrix from the reference panel), and the *y* axis represents the observed z scores (the GWAS summary statistics). Points lie along the diagonal indicating consistency between the observed value and the expected value. "s" gives information about the consistency of the LD. A larger "s" metric implies a strong inconsistency between GWAS summary statistics and the LD matrix from the reference panel.

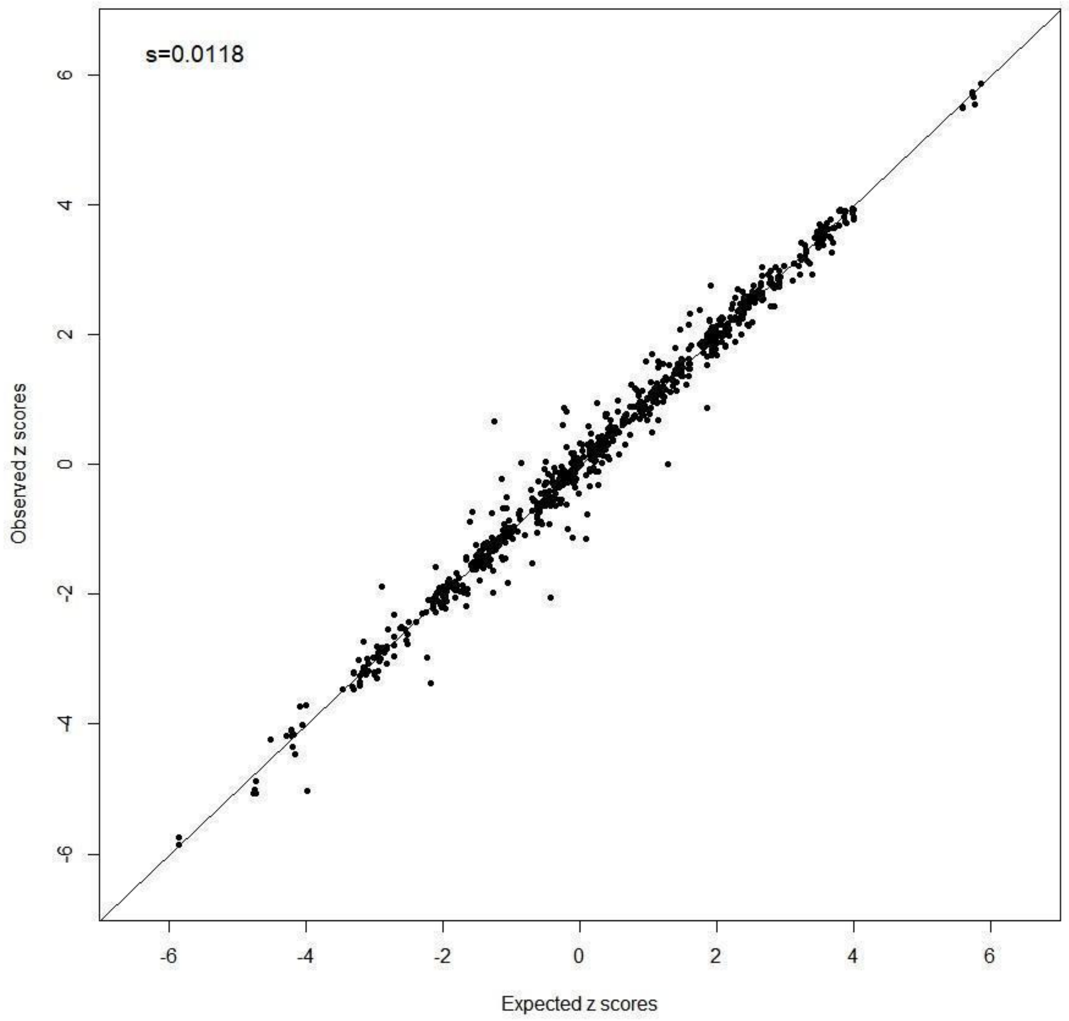

Supplementary Figure 20. Polygenic prediction of *EduYears* across discovery cohorts with consistent sample sizes in the EMCIT (*N* = 395; EAS), Korean-based cohort (N = 2,622; EAS), Chinese samples in the UKBB (*N* = 1,747; EAS) and the NIA-LOAD (*N* = 1,241; EUR). The *x* axis shows the testing cohort, and the *y* axis is the partial R^2 for the polygenic score (PGS). Bars indicate the partial R^2 for the PGS of each cohort. Error bars (black line) indicate the 95% confidence intervals of the partial R^2 for PGS. PGSs were derived from the GWAS for *EduYears* in East Asian by using PRS-CS (discovery GWAS in EAS, *N* = 176,400), the GWAS for *EduYears* in European by using PRS-CS (discovery GWAS in EUR (UKBB), *N* = 176,400 or 352,800), and both GWAS meta-analysis for *EduYears*, including East Asian and European, by using PRS-CSx (discovery GWAS in EAS and discovery GWAS in EUR (UKBB)). We adjusted for the birth year, sex, birth year by sex interaction, and top 10 principal components in all models. The two-sided *P*-value of the partial R^2 was derived from a likelihood ratio test comparing the goodness of fit of the models with and without PGS, which were annotated above the error bars.

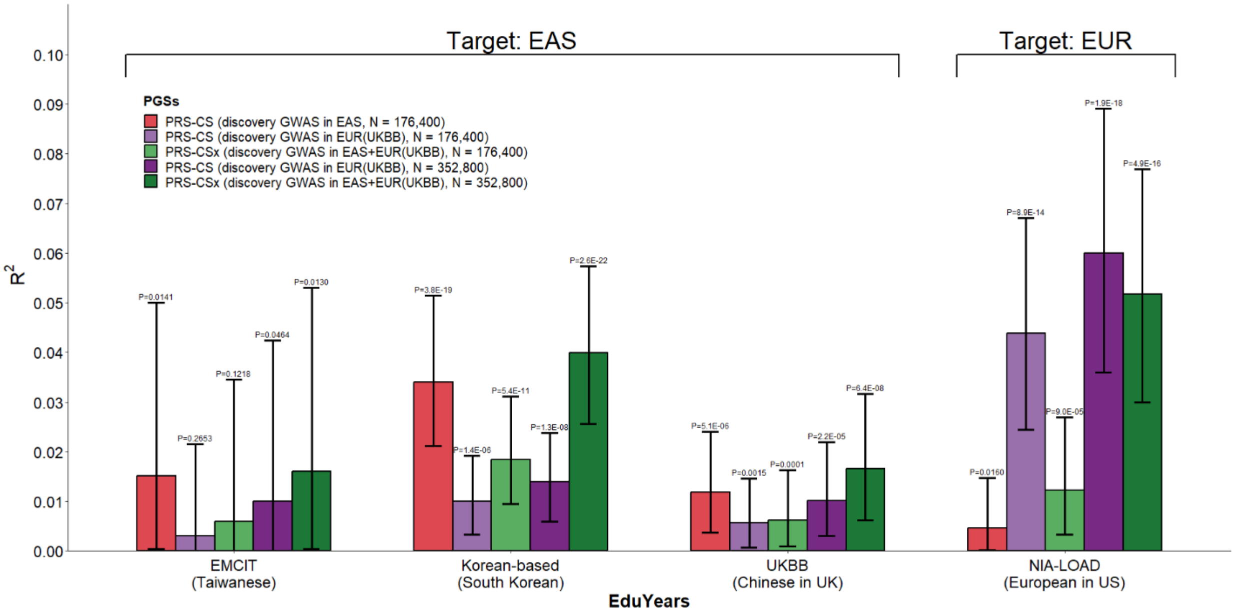

URLs

LocusZoom v0.9.6, https://my.locuszoom.org/; NHGRI-EBI GWAS Catalog, https://www.ebi.ac.uk/gwas; The Genome Aggregation Database (gnomAD), https://gnomad.broadinstitute.org; Taiwan Biobank, https://www.twbiobank.org.tw/new_web_en; PBK genotype QC project, https://github.com/Annefeng/PBK-QC-pipeline; UK Biobank quality control documentation, https://www.ukbiobank.ac.uk/wp-content/uploads/2014/04/imputation_documentation_May2015.pdf; UK Biobank, http://www.ukbiobank.ac.uk; LDSC v1.0.1, https://github.com/bulik/ldsc; GTEx, http://www.gtexportal.org/home/datasets; Franke lab data, https://data.broadinstitute.org/mpg/depict/depict_download/tissue_expression; Cahoy *et al*. data, https://www.jneurosci.org/content/suppl/2008/01/03/28.1.264.DC1; KoGES PheWeb, https://koges.leelabsg.org; Regenie v2.2.4, https://rgcgithub.github.io/regenie/; PLINK v2.0,https://www.cog-genomics.org/plink/2.0/; METAL 2020-05-05, https://github.com/statgen/METAL; SnpSift v4_3t_core, https://pcingola.github.io/SnpEff/; S-LDXR v0.3-beta, https://huwenboshi.github.io/s-ldxr/; FUMA v1.3.7, https://fuma.ctglab.nl/; SuSiEx v1.0.0, https://github.com/getian107/SuSiEx/; R v4.2.1, https://www.r-project.org/; susieR package v0.12.10, https://stephenslab.github.io/susieR/index.html; PRS-CS v1.0.0, https://github.com/getian107/PRScs; PRS-CSx v1.0.0, https://github.com/getian107/PRScsx/.

Supplementary References

1. Current education system from Ministry of Education in Taiwan: https://english.moe.gov.tw/cp-126-17722-3fb83-1.html
2. Implementation scheme for 12-year basic education from Ministry of Education in Taiwan: https://english.moe.gov.tw/cp-48-23066-05c85-1.html
3. Study in Korea run by Korean Government: https://www.studyinkorea.go.kr/en/overseas_info/allnew_higherEducation.do
4. Ministry of Public Administration and Security National Archives of Korea: https://theme.archives.go.kr/next/koreaOfRecord/compulsoryEdu.do
5. Werme, J., van der Sluis, S., Posthuma, D. & de Leeuw, C. A. An integrated framework for local genetic correlation analysis. *Nature genetics* **54**, 274-282 (2022).
6. Chuang, Y. F. *et al.* Urban-rural differences in the prevalence and correlates of mild cognitive impairment in community-dwelling older adults in Taiwan: The EMCIT study. *J Formos Med Assoc* **120**, 1749-1757 (2021).
7. Jung, S.-H. *et al.* Transferability of Alzheimer Disease Polygenic Risk Score Across Populations and Its Association With Alzheimer Disease-Related Phenotypes. *JAMA Network Open* **5**, e2247162-e2247162 (2022).
8. Privé, F. *et al.* Portability of 245 polygenic scores when derived from the UK Biobank and applied to 9 ancestry groups from the same cohort. *The American Journal of Human Genetics* **109**, 12-23 (2022).
9. Kuzma, A. *et al.* NIAGADS: the NIA genetics of Alzheimer's disease data storage site

(Wiley Online Library, 2016).
10. Naj, A. C. *et al.* Common variants at MS4A4/MS4A6E, CD2AP, CD33 and EPHA1 are associated with late-onset Alzheimer's disease. *Nature genetics* **43**, 436-441 (2011).
11. Ge, T., Chen, C.-Y., Ni, Y., Feng, Y.-C. A. & Smoller, J. W. Polygenic prediction via Bayesian regression and continuous shrinkage priors. *Nature communications* **10**, 1-10 (2019).
12. Ruan, Y. *et al.* Improving polygenic prediction in ancestrally diverse populations. *Nature Genetics* **54**, 573-580 (2022).
