# Supplementary material for: Shared genetic architectures of educational attainment in East Asian and European populations
Source: Nat Hum Behav. 2024 Jan 5;8(3):562–75. doi: 10.1038/s41562-023-01781-9 (PMC10963262; doi:10.1038/s41562-023-01781-9)
Supplement: Supplementary file 2 — Reporting Summary [file 41562_2023_1781_MOESM2_ESM.pdf]

nature portfolio

Corresponding author(s): Yen-Feng Lin, Woojae Myung, Chia-Yen Chen, Hong-Hee Won

Last updated by author(s): Oct 30, 2023

Reporting Summary

Nature Portfolio wishes to improve the reproducibility of the work that we publish. This form provides structure for consistency and transparency in reporting. For further information on Nature Portfolio policies, see our Editorial Policies and the Editorial Policy Checklist.

Statistics

For all statistical analyses, confirm that the following items are present in the figure legend, table legend, main text, or Methods section.

n/a	Confirmed	
☐	☒	The exact sample size (*n*) for each experimental group/condition, given as a discrete number and unit of measurement
☐	☒	A statement on whether measurements were taken from distinct samples or whether the same sample was measured repeatedly
☐	☒	The statistical test(s) used AND whether they are one- or two-sided<br>*Only common tests should be described solely by name; describe more complex techniques in the Methods section.*
☐	☒	A description of all covariates tested
☐	☒	A description of any assumptions or corrections, such as tests of normality and adjustment for multiple comparisons
☐	☒	A full description of the statistical parameters including central tendency (e.g. means) or other basic estimates (e.g. regression coefficient) AND variation (e.g. standard deviation) or associated estimates of uncertainty (e.g. confidence intervals)
☐	☒	For null hypothesis testing, the test statistic (e.g. *F*, *t*, *r*) with confidence intervals, effect sizes, degrees of freedom and *P* value noted<br>*Give P values as exact values whenever suitable.*
☐	☒	For Bayesian analysis, information on the choice of priors and Markov chain Monte Carlo settings
☒	☐	For hierarchical and complex designs, identification of the appropriate level for tests and full reporting of outcomes
☐	☒	Estimates of effect sizes (e.g. Cohen's *d*, Pearson's *r*), indicating how they were calculated

Our web collection on statistics for biologists contains articles on many of the points above.

Software and code

Policy information about availability of computer code

Data collection	No software was used for data collection.
Data analysis	Previously developed pipelines were used to produce the results of the current study. No custom code was developed. LocusZoom v0.9.6, https://my.locuszoom.org/; PBK genotype QC project, https://github.com/Annefeng/PBK-QC-pipeline; UK Biobank quality control documentation, https://www.ukbiobank.ac.uk/wp-content/uploads/2014/04/imputation_documentation_May2015.pdf; LDSC v1.0.1, https://github.com/bulik/ldsc; Regenie v2.2.4, https://rgcgithub.github.io/regenie/; PLINK v2.0,https://www.cog-genomics.org/plink/2.0/; METAL 2020-05-05, https://github.com/statgen/METAL; SnpSift v4_3t_core, https://pcingola.github.io/SnpEff/; S-LDXR v0.3-beta, https://huwenboshi.github.io/s-ldxr/; FUMA v1.3.7, https://fuma.ctglab.nl/; SuSiEx v1.0.0, https://github.com/getian107/SuSiEx/; R v4.2.1, https://www.r-project.org/; susieR package v0.12.10, https://stephenslab.github.io/susieR/index.html; PRS-CS v1.0.0, https://github.com/getian107/PRScs; PRS-CSx v1.0.0, https://github.com/getian107/PRScsx/.

For manuscripts utilizing custom algorithms or software that are central to the research but not yet described in published literature, software must be made available to editors and reviewers. We strongly encourage code deposition in a community repository (e.g. GitHub). See the Nature Portfolio guidelines for submitting code & software for further information.

Data

Policy information about availability of data

All manuscripts must include a data availability statement. This statement should provide the following information, where applicable:

- Accession codes, unique identifiers, or web links for publicly available datasets
- A description of any restrictions on data availability
- For clinical datasets or third party data, please ensure that the statement adheres to our policy

A detailed description of the availability and application process of the individual-level TWB data can be found at https://www.biobank.org.tw/english.php. Briefly, TWB made available the individual-level data and biological samples from the participants of the prospective cohort study in 2014. Available data include questionnaire surveys, physical measures, blood and urine tests, biological samples and genomic data (whole-genome sequencing, whole-genome genotyping, DNA methylation, HLA typing, and blood metabolome). Researchers interested in obtaining TWB individual-level data for research purposes would need to submit an application that includes a detailed research proposal and an institutional review board (IRB) approval to TWB (contact email: biobank@gate.sinica.edu.tw). The application will undergo scientific and ethical reviews by external experts in relevant scientific fields and the TWB ethical governance committee (EGC). Once approved, researchers will be able to access the data for the approved research projects during the approved time period. For international researchers outside of Taiwan, an additional international data transfer agreement needs to be filed to the Ministry of Health and Welfare of Taiwan to enable sharing of the TWB individual-level data and any derived data. Access to KoGES data, including phenotypes and genotypes, is granted upon approval from the Institutional Review Board of the Korean National Institute of Health. Comprehensive details on KoGES data distribution can be found at the Korea Biobank Project website (https://www.kdca.go.kr/contents.es?mid=a30326000000). Data from the UKBB is available on application to their site (UK Biobank, https://www.ukbiobank.ac.uk). Data from the NIA-LOAD can be accessed from dbGaP under accession number (phs000168.v1.p1). Summary statistics of EUR GWAS for EduYears by Lee and colleagues are publicly available at the Social Science Genetic Association Consortium (SSGAC, https://www.thessgac.org/). The full summary statistics of EAS GWAS and cross-ancestry GWAS are publicly available at the NHGRI-EBI GWAS Catalog (https://www.ebi.ac.uk/gwas/downloads) with accession numbers GCST90296498 and GCST90296499, respectively.

Human research participants

Policy information about studies involving human research participants and Sex and Gender in Research.

Reporting on sex and gender	Sex was used as a covariate in the GWAS of educational attainment (EduYears) in East Asian. In total, 63,531 males (36%) and 112,879 (64%) females were included in the East Asian GWAS.
Population characteristics	For meta-analysis in East Asian population:<br>104,722 individuals (37,766 males, the birth year ranged from 1939-1989) in Taiwan Biobank (TWB) and 71,678 individuals (25,755 males, the birth year ranged from 1918-1973) in Korean Genome and Epidemiology Study (KoGES). More population characteristics were described in Supplementary Table 1.
Recruitment	Researchers in this study were not involved in the participant recruitment.
Ethics oversight	This study has been approved by the ethics committee of National Health Research Institutes, Taiwan (TWB; EC1090402-E and EC1110608-E) and Seoul National University Bundang Hospital, South Korea (KoGES; X-2107-699-902).

Note that full information on the approval of the study protocol must also be provided in the manuscript.

Field-specific reporting

Please select the one below that is the best fit for your research. If you are not sure, read the appropriate sections before making your selection.

☐ Life sciences ☒ Behavioural & social sciences ☐ Ecological, evolutionary & environmental sciences

For a reference copy of the document with all sections, see nature.com/documents/nr-reporting-summary-flat.pdf

Behavioural & social sciences study design

All studies must disclose on these points even when the disclosure is negative.

Study description	This is a genome-wide association study (GWAS) for EduYears in East Asian population, followed by a cross-population GWAS meta-analysis for EduYears between East Asian and European populations. The phenotype data is quantitative.
Research sample	The total sample size was 942,745, consisting of 176,400 East Asian samples from two nation-wide biobanks (TWB and KoGES) and 766,345 individuals of European genome-wide summary statistics. Both TWB and KoGES are population-based prospective cohorts by recruiting volunteers. The mean age was 49.9 ± 10.9 years for TWB samples and 54.1 ± 8.3 years for KoGES samples. There were more females than males in both cohorts. Please find the detail information for TWB and KoGES in Supplementary Table 1. In this study, we present the largest-to-date EduYears GWAS in East Asian population and cross-ancestry GWAS meta-analysis across East Asian and European populations for EduYears.

Sampling strategy	Both TWB and KoGES are population-based prospective cohorts by recruiting volunteers. We did not perform sample size calculation; instead, we conducted an international collaborative study to maximize the sample size of East Asian for GWAS on educational attainment.
Data collection	In this observational study, data was collected independently in each cohort. Baseline characteristic data were collected from interviews, physical measurements, biomarkers, and genetic data in both TWB and KoGES. Blinding is not applicable as the paper reports a genome-wide association study for an observed variable with no experimental manipulation.
Timing	TWB recruited participants from 2012 to 2020, and KoGES recruited participants from 2001 to 2013.
Data exclusions	After stringent quality control (QC), we excluded 2,771 samples from TWB and 616 samples from KoGES in the EAS GWAS.
Non-participation	No participants dropped out or declined participation.
Randomization	This genome-wide association study (GWAS) is an observational study. Randomization is not applicable in this study.

Reporting for specific materials, systems and methods

We require information from authors about some types of materials, experimental systems and methods used in many studies. Here, indicate whether each material, system or method listed is relevant to your study. If you are not sure if a list item applies to your research, read the appropriate section before selecting a response.

Materials & experimental systems

n/a	Involved in the study	
☒	☐	Antibodies
☒	☐	Eukaryotic cell lines
☒	☐	Palaeontology and archaeology
☒	☐	Animals and other organisms
☒	☐	Clinical data
☒	☐	Dual use research of concern

Methods

n/a	Involved in the study	
☒	☐	ChIP-seq
☒	☐	Flow cytometry
☒	☐	MRI-based neuroimaging
